# Clustering on longitudinal lifestyle trajectories and their impact on cognitive performance

**DOI:** 10.3389/fpsyg.2025.1510971

**Published:** 2025-07-25

**Authors:** Alba Roca-Ventura, Javier Solana-Sánchez, Gabriele Cattaneo, Vanessa Alviarez-Schulze, Goretti España-Irla, María Redondo-Camós, Selma Delgado-Gallén, Ruben Romero, Edgar Buloz-Osorio, Álvaro Pascual-Leone, David Bartrés-Faz

**Affiliations:** ^1^Institut Guttmann, Institut Universitari de Neurorehabilitació adscrit a la UAB, Badalona, Spain; ^2^Departament de Medicina, Facultat de Medicina i Ciències de la Salut, i Institut de Neurociències, Universitat de Barcelona, Barcelona, Spain; ^3^Fundació Institut d’Investigació en Ciències de la Salut Germans Trias i Pujol, Barcelona, Spain; ^4^Universitat Autònoma de Barcelona, Bellaterra, Spain; ^5^Hinda and Arthur Marcus Institute for Aging Research and Deanna and Sidney Wolk Center for Memory Health, Hebrew SeniorLife, Boston, MA, United States; ^6^Department of Neurology, Harvard Medical School, Boston, MA, United States; ^7^Institut d’Investigacions Biomèdiques August Pi I Sunyer (IDIBAPS), Barcelona, Spain

**Keywords:** lifestyle and behavior, clustering analysis, brain health, longitudinal analyses, cognition, neuropsychological assessment

## Abstract

**Introduction:**

Lifestyle factors have demonstrated a significant contribution to resilience against cognitive decline and age-related diseases. However, the understanding of how combinations of modifiable lifestyle behaviors relate to cognitive trajectories across the lifespan remains limited. This study aims to explore the relationship between lifestyle trajectories—including cognitive activity, physical exercise, sleep, socialization, nutrition, alcohol consumption, tobacco use, and body mass index (BMI)—and cognitive performance in healthy middle-aged adults.

**Methods:**

Data were obtained from the Barcelona Brain Health Initiative (BBHI), an ongoing longitudinal prospective cohort study. Participants completed repeated self-reports on lifestyle factors and underwent in-person neuropsychological assessments. Kml3d clustering was applied to the longitudinal lifestyle data to identify distinct profiles. Cognitive performance was then analyzed across these lifestyle clusters to evaluate associations between lifestyle patterns and cognitive status.

**Results:**

The results revealed that adherence to healthy lifestyle patterns was strongly associated with better cognitive performance. Specifically, individuals following profiles characterized by higher engagement in cognitive and physical activities, healthier nutrition, better psychological health, and stronger socialization showed superior cognitive status. Moreover, the findings underscored that adhering to a higher number of healthy behaviors had a cumulative positive impact on cognition. Across the studied period—spanning middle age to older adulthood—cognitive trajectories were generally stable.

**Discussion:**

This study highlights that k-means clustering of longitudinal lifestyle data can successfully identify meaningful lifestyle profiles associated with cognitive status in middle-aged adults. The results suggest that specific combinations of modifiable lifestyle factors may exert a more pronounced influence on maintaining cognitive health. These findings provide promising insights for the development and personalization of lifestyle interventions aimed at enhancing brain health and resilience to cognitive decline.

## Introduction

The continuous advancements in public health policies and medical innovations have led to a notable extension in human life expectancy. In this regard, current projections indicate that the population of adults aged 65 and above is predicted to be more than double by the year 2050. Nevertheless, the growing population also represents an increasing prevalence of age-related diseases, presenting significant challenges to both socioeconomic and healthcare systems (Harper, 2014). One of the major concerns of aging is cognitive decline, which can lead to disability and dependency, and which is frequently associated with cerebrovascular disorders and neurodegenerative diseases. However, the central question has been why some individuals succeed in aging successfully, retaining their ability to perform physical and mental functions as well as maintaining emotional stability and social interaction, while others experience cognitive decline (Rowe and Kahn, 2015).

The reserve model offers a useful framework to explain individual differences in cognitive aging (Stern, 2002). According to this framework, cognitive deficits typically emerge when the reserve of an individual—whether structural (brain reserve) or functional (cognitive reserve)—falls below a critical threshold. Individuals with lower baseline reserve are more susceptible to exhibiting clinical symptoms earlier, as they have fewer resources to compensate for the effects of aging and pathological changes (Katzman et al., 1988). In contrast, those with higher reserve levels can maintain cognitive functioning for a longer period, as their greater resource capacity provides a buffer against these detrimental influences (Fritsch et al., 2007; Stern et al., 2023). Brain reserve refers to a passive model, where structural characteristics such as brain size or neuronal count, determine the capacity of the brain to tolerate pathology. Cognitive reserve, by contrast, represents an active model that emphasizes the flexible and efficient use of neural networks to compensate for damage (Arenaza-Urquijo et al., 2015). While the distinction between these two forms of reserve is largely conceptual, they interact closely. Functional adaptability (cognitive reserve) is often supported by anatomical resources (brain reserve), highlighting the intertwined nature of structure and function in sustaining cognitive health (Stern, 2009). Even during typical aging, structural brain changes—such as cortical thinning, white matter and gyral atrophy, ventricular enlargement (Blinkouskaya et al., 2021), and hippocampal volume loss (Bettio et al., 2017)—have been associated with declines in various cognitive domains, including executive functions, processing speed (Prins et al., 2005), episodic memory (Lalla et al., 2022), and working memory (Bartrés-Faz et al., 2009; Pliatsikas et al., 2019).

Initial reserve levels are shaped by genetic and developmental factors, but they can also be modified throughout life by environmental exposures and experiences—from childhood through older age (Stern et al., 2020, 2023). Since there are no direct measures of reserve, researchers commonly rely on proxy indicators. For brain reserve, typical proxies include intracranial volume, brain volume, and head circumference, whereas education, IQ, occupational complexity, and other cognitive activities are widely used proxies for cognitive reserve (Arenaza-Urquijo et al., 2015). However, a broader range of modifiable lifestyle factors—such as physical activity, cognitive engagement, social interaction, and dietary habits—also play a significant role in influencing reserve capacity (Tucker and Stern, 2011; Koščak Tivadar, 2017; Liu et al., 2020; Wu et al., 2020; Oosterhuis et al., 2022; Song et al., 2022; Abellaneda-Pérez et al., 2023). These factors not only contribute to cognitive reserve but have also been extensively studied for their role in mitigating cognitive decline (Amanollahi et al., 2021) and delaying the onset of neurodegenerative diseases—potentially accounting for up to 45% of dementia cases (Xu et al., 2020; Livingston et al., 2024). Importantly, lifestyle behaviors may influence both brain structure and brain function, thereby modulating both brain reserve (passive model) and cognitive reserve (active model) (Bartrés-Faz et al., 2009; Arenaza-Urquijo et al., 2013).

Understanding the complexity of lifestyle habits is essential, as growing evidence suggests that combinations of behaviors—rather than individual factors in isolation—are better reserve proxies and may play a critical role in explaining the substantial inter-individual variability observed in cognitive aging (Cockerham, 2005; Šneidere et al., 2024). However, current approaches often simplify lifestyle assessments, treating lifestyle factors as mere components in a simplistic equation without considering their complex interactions and synergistic effects. A common method involves constructing composite indices by summing up the individual impacts of various health behaviors, operating under the assumption of their interchangeable significance (Loef and Walach, 2012; McAloney et al., 2013; Bittner et al., 2019, 2021; Franz et al., 2021). This approach fails to account for the interconnected nature of lifestyle behaviors, which tend to aggregate into distinct patterns within the population based on shared characteristics or underlying factors (Noble et al., 2015).

To address these limitations, unsupervised clustering techniques offer a valuable alternative by uncovering naturally occurring patterns in lifestyle data without relying on predefined categories. These methods allow researchers to explore how lifestyle behaviors co-occur and influence one another, providing a more nuanced view of their combined effects. By revealing these hidden patterns, clustering approaches help clarify the dynamic and interactive nature of lifestyle behaviors, contributing to a more comprehensive understanding of their impact on health and wellbeing.

Moreover, non-supervised data analyses based on self-reported online assessments over time allow exploration of trajectory patterns in longitudinal lifestyle data in a very efficient manner. By tracking changes in lifestyle behaviors over time within individual participants or population subgroups, researchers can uncover distinct trajectories of behavior evolution and identify factors that influence these trajectories. This longitudinal perspective offers valuable insights into the dynamic nature of lifestyle patterns and informs the development of targeted interventions tailored to individual needs and preferences.

In our previous investigation (Roca-Ventura et al., 2024), the KmL3D (Genolini et al., 2013), a longitudinal clustering method, was employed to examine the joint evolution of nine modifiable health behaviors known to influence brain health (Cattaneo et al., 2018; Livingston et al., 2024)—cognitive activity, physical activity, nutrition, sleep, socialization, obesity (BMI), alcohol intake, tobacco use, and vital plan—in a sample of 3,013 middle-aged adults from the Barcelona Brain Health Initiative (Cattaneo et al., 2018). This technique allowed the identification of five distinct lifestyle profiles, each associated with specific disease risks. The “Healthy” cluster included individuals who consistently maintained high engagement in cognitive, physical, and social activities, a healthy diet, good sleep, and low-risk behaviors such as minimal alcohol and tobacco use, and normal BMI; this group showed the lowest incidence of chronic diseases and the most favorable health indicators. The “Low Cognitive Reserve” cluster was characterized by low cognitive engagement, socialization, and purpose in life, despite relatively healthy values in other domains; this profile was associated with increased risk of neurological conditions and lower mental wellbeing. The “Obesogenic” cluster reflected high BMI, poor diet and exercise habits, leading to greater cardiometabolic risk and multimorbidity. The “Heavy Smokers” cluster, characterized by high tobacco use, showed increased vulnerability to cardiovascular and neurological diseases. Finally, the “Alcohol-Sleep” cluster, was defined by harmful alcohol intake, poor sleep quality, and low wellbeing, showing the highest rates of psychiatric disorders and negative self-rated health. These lifestyle patterns remained stable over the study period and were predictive of disease risk.

This study proposes that the extracted longitudinal lifestyle clusters are useful for exploring the impact of lifestyle choices on cognitive aging in middle-aged adults. This approach enables the crucial tracking of behavioral change patterns over time as subjects transition from middle age to older adulthood. Recognizing and comprehending these lifestyle factors during middle age is crucial, as modifiable risk factors for dementia can exert their influence years before symptoms become evident (Hughes and Ganguli, 2009; Kesse-Guyot et al., 2014; Artaud et al., 2016). Hence, addressing these factors preemptively holds the potential to mitigate the risk of cognitive decline and promote enduring brain health.

In this context, the objectives of this study are twofold: first, to validate the use of unsupervised methods in lifestyle clustering trajectories in relation to cognitive performance; and second, to examine the differential evolution of cognition over time within these identified profiles.

## Materials and methods

### Participants and study design

**Figure 1:**
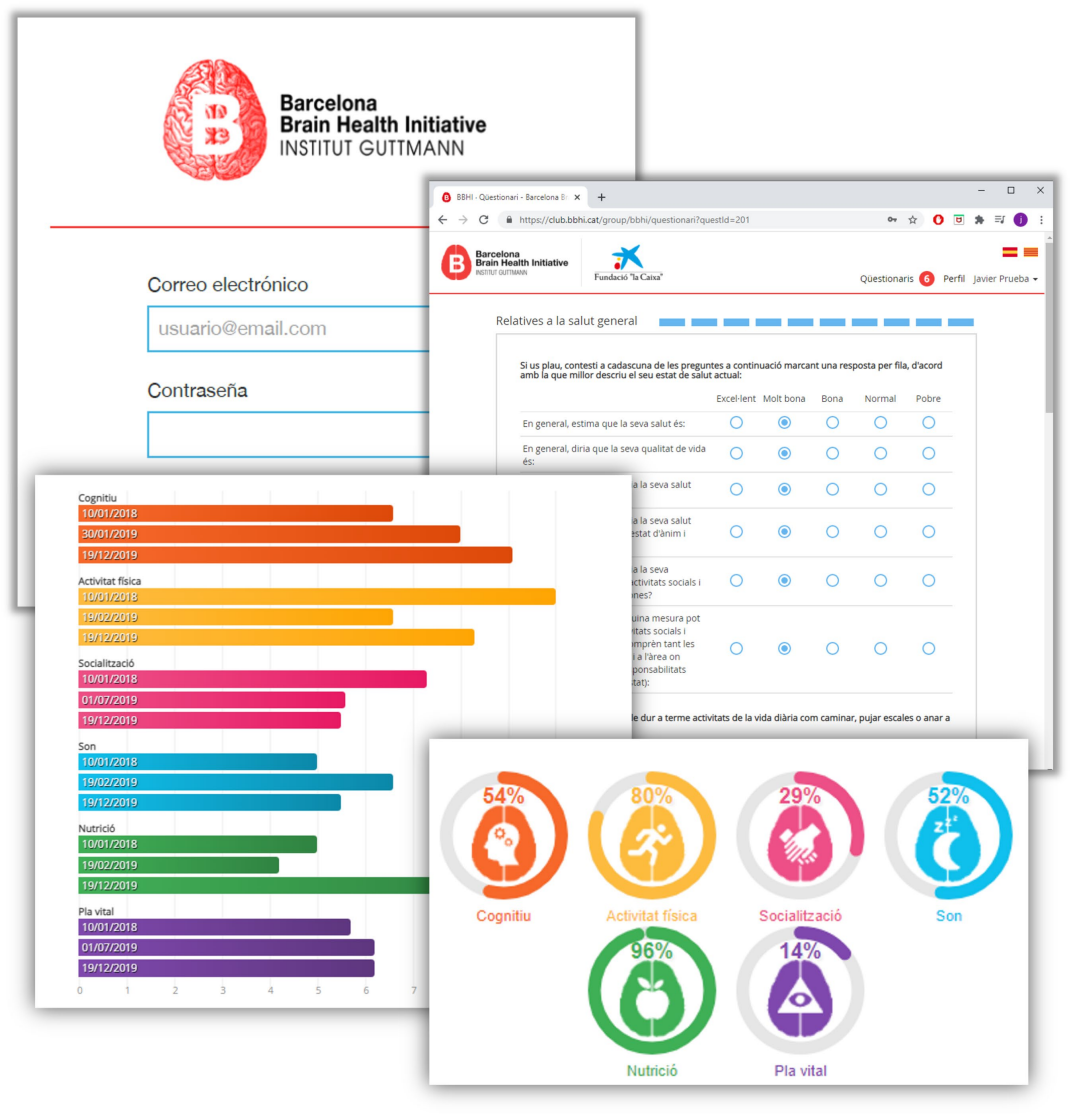
Overview of the BBHI web-based platform, showcasing the log-in page, an example of the online questionnaire, and the feedback profiles generated by the platform. The figure illustrates how participants access the platform, complete the lifestyle and health-related questionnaires, and receive personalized feedback profiles based on their responses.

**Figure 2:**
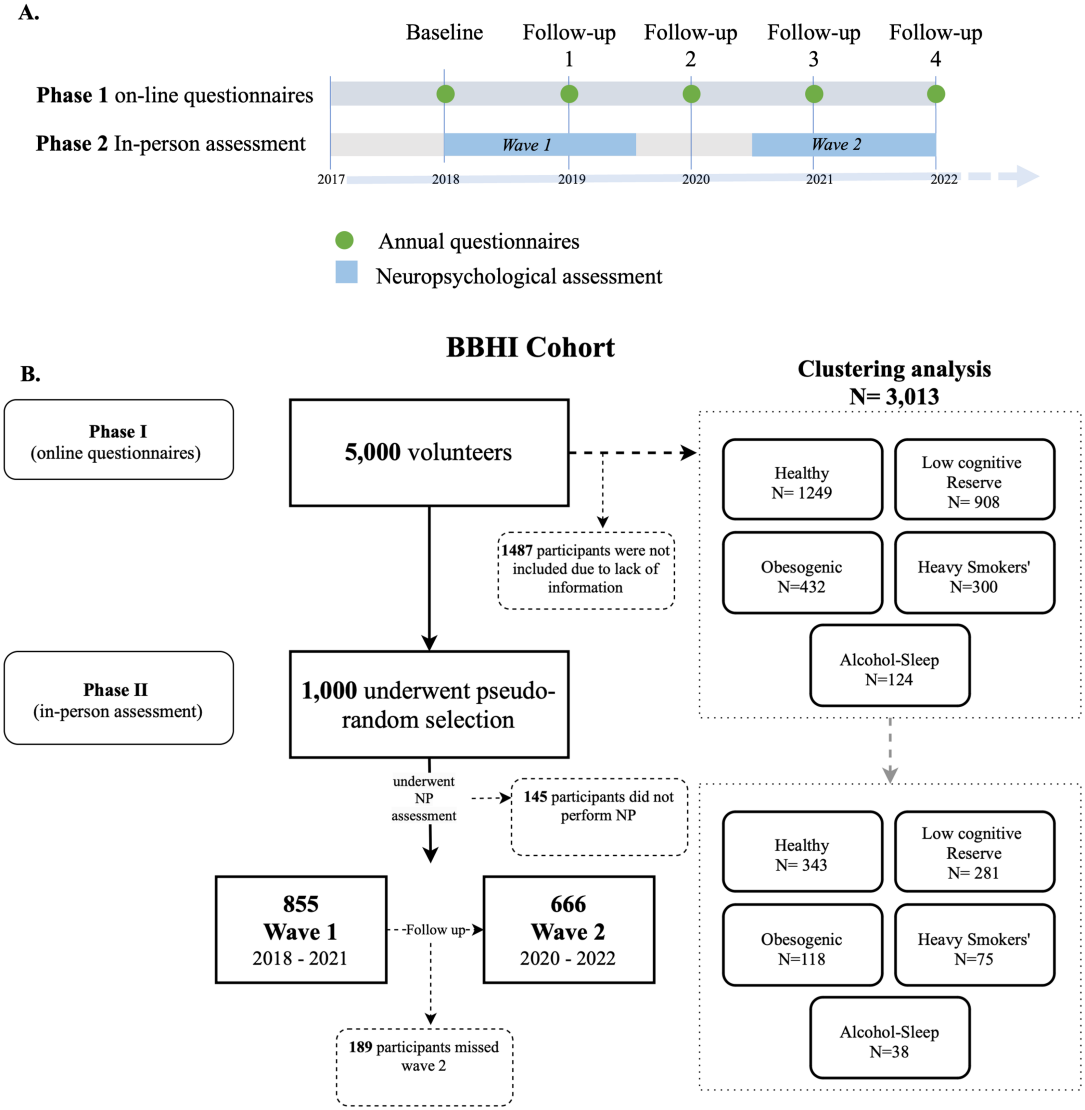
**(A)** Timeline of the Barcelona Brain Health Initiative. **(B)** Flowchart of the selection and distribution of participants in the study.

This study was conducted within the Barcelona Brain Health Initiative (BBHI) framework (Cattaneo et al., 2018; Cattaneo et al., 2020), an ongoing prospective longitudinal study launched in 2017 aimed at identifying lifestyle and biological factors influencing brain health in middle-aged adults. From 2018 to 2022, over 5,000 participants completed Phase 1, which involved an annual online self-assessment via the BBHI web-based platform (Figure 1). These assessments collected data on sociodemographic characteristics, lifestyle habits, medical history, and personality traits. See Figure 2 for the study design.

In parallel, between May 2018 and February 2021 (Wave 1) and April 2020 and December 2022 (Wave 2), a subgroup of 1,000 participants from the overall cohort were invited to participate in an in-depth, in-person assessment (Phase 2) in the Guttmann Institute Centre in Barcelona, Spain. This phase included a comprehensive neuropsychological evaluation conducted in a single session by licensed neuropsychologists (AR, VA) (Figure 2), applying a consistent evaluation protocol across all participants.

**Figure 3:**
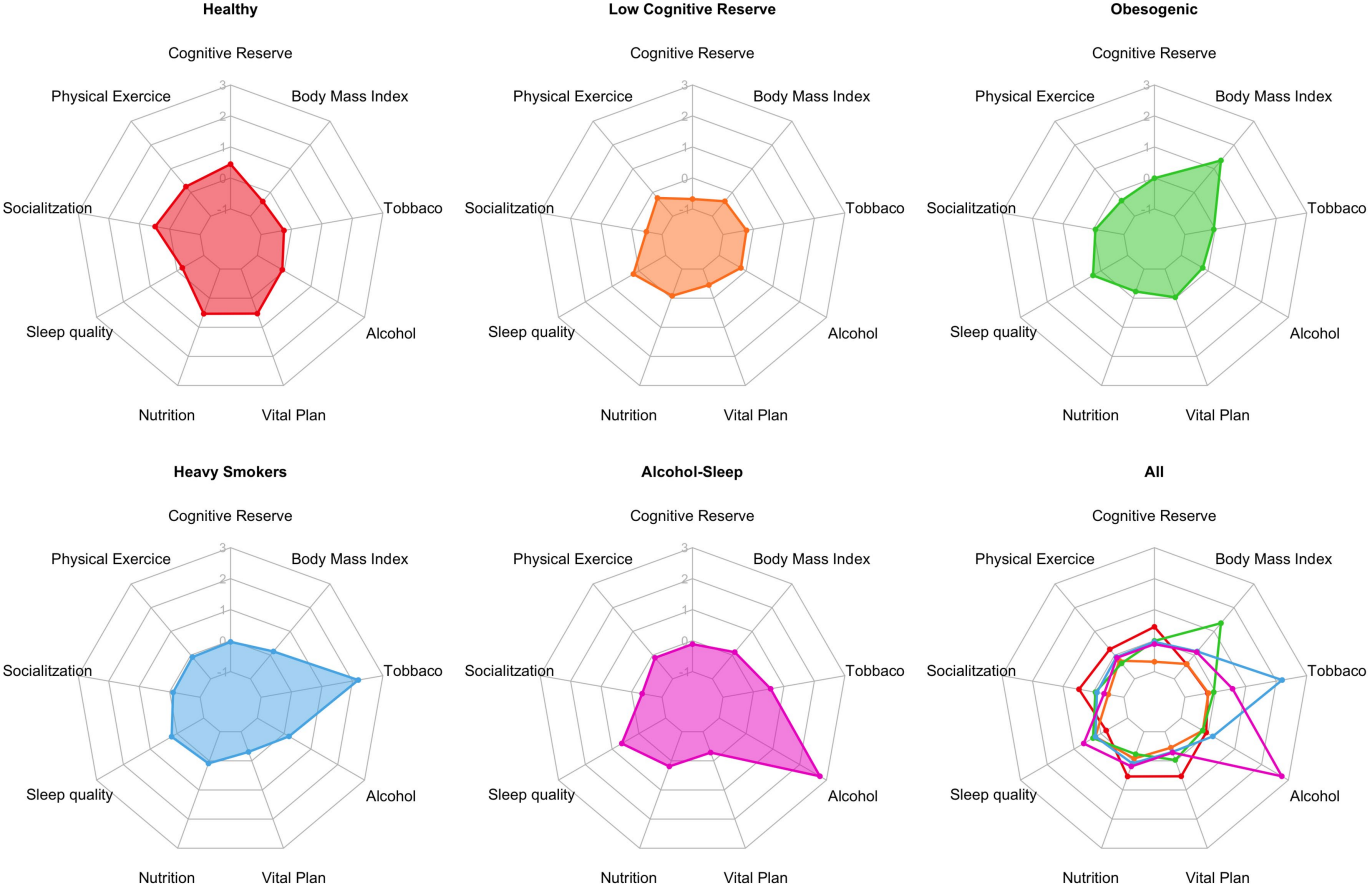
Radar plots showing the normalized distribution of the modifiable risk factors in each cluster (*n* = 3,013).

Roca-Ventura et al. (2024) identified five distinct lifestyle profiles among 3,013 BBHI participants using a data-driven analysis of online follow-up questionnaires from Phase 1. These clusters were labeled based on their most prevalent risk factors: Healthy (*n* = 1,249), Low cognitive reserve (*n* = 908), Obesogenic (*n* = 432), Heavy Smokers (*n* = 300) and Alcohol-Sleep (*n* = 124) (Figure 3).

In the present study, we introduce a novel aspect by utilizing data from the in-person comprehensive neuropsychological assessments conducted in Phase 2, unlike the prior study that focused on online self-assessments from Phase I. In-depth evaluations offer a deeper understanding of cognitive functioning. A total of 855 participants from the identified clusters completed the neuropsychological assessments in Wave 1, and 666 of these participants were reassessed in Wave 2, providing a unique opportunity to explore cognitive profiles in relation to previously identified lifestyle factors. All participants scored above 26 points on the Mini-Mental State Examination (MMSE), suggesting a normal general cognition performance. This criterion was used to ensure that the sample did not include individuals with global cognitive impairment at baseline.

All participants gave written informed consent to the study which was approved by the ethics and research committee of the Institut Guttmann (Badalona, Spain) and complied with the recommendations of the “Unió Catalana d’Hospitals.”

### Cognitive assessment

Results from two timepoints (wave 1 and wave 2) were collected. Neuropsychological assessment consisted of a battery of well-established neuropsychological tests, exploring general/fluid intelligence (Wechsler Adult Intelligence Scale IV [WAIS-IV] Matrix Reasoning; Weschler, 2008), premorbid intelligence (TAP-30, Pluck et al., 2017) and global cognitive status, screened using Mini-Mental State Examination (MMSE; Folstein et al., 1975), semantic verbal fluency (Peña-Casanova, 2005), associative memory (Spanish Version of the Face Name Associative Memory Exam S-FNAME, Alegret et al., 2015; Alviarez-Schulze et al., 2022), visuospatial searching, selective attention, visual/motor and processing speed (Cancelation test and digit symbol substitution from WAIS-IV; Weschler, 2008), Trail Making Test-A (TMT-A, Reitan and Wolfson, 1985), cognitive flexibility and set-shifting (Trial making test B [TMT-B]; Reitan and Wolfson, 1985), phonemic verbal fluency (Peña-Casanova, 2005), working memory (Digit Forward, Digit Backward and Letter number sequencing; Weschler, 2008), episodic memory (Rey Auditory Verbal Learning Test [RAVLT]; Rey, 1958), visuo-spatial abilities (Block design, Weschler, 2008). Following the same procedure used in previous studies, z-scores were calculated for each test, and a composite score was created by taking the average of all the z-score ratings together, resulting in a single score that represents global cognitive performance (España-Irla et al., 2021; Cattaneo et al., 2022). Positive z scores indicate better cognitive performance, whereas negative z scores indicate poorer cognitive performance relative to the mean performance of participants at baseline.

Three cognitive domains were also calculated by taking the average of z-scores tasks for memory, executive functioning, and processing speed. The memory domain included S-FNAME, immediate memory, delayed recall, and recognition of RAVLT. The executive functioning domain included phonemic verbal fluency, Trail Making Test B, digital span backward, and letter number sequencing. The processing speed domain included digit symbol substitution. These three domains have been found to be representative of cognitive decline in other studies (Ngandu et al., 2015), and the tasks in each domain were selected by an experienced neuropsychologist.

### Statistical analyses

Statistical analyses were conducted with using R Studio (version 2022.07.1+554) with R version 4.2.2 (2022-10-31), with significance set at *p* < 0.05. The KmL3D package was employed to apply k-means clustering to longitudinal data (Genolini et al., 2013) targeting the joint trajectories of nine lifestyle behaviors associated with brain health (Cattaneo et al., 2018; Livingston et al., 2024)—cognitive activity, physical activity, nutrition, sleep, socialization, obesity (BMI), alcohol intake, tobacco use, and vital plan. Full definitions and the instruments used to assess these behaviors are provided in Supplementary material and in Roca-Ventura et al. (2024).

This unsupervised machine learning technique serves to condense diverse longitudinal data into coherent, uniform clusters. Other methods analyze each variable-trajectory independently and then consider the combination of partitions obtained, but these approaches do not enable detection of groups where the co-evolution of the variables is complex.

Determining the optimal number of clusters was accomplished through non-parametric computations employing the Calinski-Harabasz index, which evaluates both between-cluster and within-cluster variances (Genolini et al., 2015). Each observation denoting individual’s average composite lifestyles score across 2018–2022 was assigned to one of five clusters. Next, each participant was reassigned iteratively to the closest cluster, yielding five joint developmental trajectories. Participants were assigned to the closest trajectory based on the data available, with missing data handled by the CopyMean imputation method for participants with data for at least two time points. CopyMean first uses the Last Occurrence Carried Forward (LOCF) method to obtain an approximation of the imputed value, and then uses the population mean trajectory to refine the first approximation. KmL3D ran 500 times with different initial assignments to find the best cluster solution (Sugar and James, 2003; Hand and Krzanowski, 2005; Genolini et al., 2015; Chow et al., 2023). The whole process was repeated with the number of clusters (i.e., trajectories) ranging from 2 to 5, to identify the optimal number of trajectories. We selected the best solution based on multiple model-fitting criteria (see Appendix S1 for details).

Associations between clusters and cognitive performance were analyzed using a Linear Mixed-Effects Model (LMM) with fixed effects for time and clusters, their interaction, and a random intercept for subjects to account for individual variability. Despite some deviation from normality (assessed via Shapiro–Wilk test), Q–Q plots supported approximate normality. Given the unequal group sizes, we adopted the Kruskal-Wallis test for comparisons, supported by Levene’s test (homogeneity of variance assumed) and Mauchly’s test for sphericity where applicable. The analysis encompassed cognitive composite score and cognitive domains (executive functioning, processing speed and memory). The subtests were classified in these three domains by a certified neuropsychologist. As the Cognitive Reserve Questionnaire (CRQ) was used as one of the input variables in the clustering process, and includes components such as educational level and occupational complexity, these factors are already partially embedded in the definition of lifestyle clusters. Supporting this, CRQ alone explained 9.6% of the variance in education (*R*^2^ = 0.096, *p* < 0.001) and 6.0% of the variance in occupational level (*R*^2^ = 0.060, *p* < 0.001). Adding cluster membership to models that already included CRQ only improved model fit marginally (Δ*R*^2^ ≈ 1.1% for education and Δ*R*^2^ ≈ 0.7% for occupation; both *p* < 0.001), indicating that most of the educational and occupational variance across clusters was already captured by CRQ. Therefore, we did not adjust for education or occupation in the primary regression models, to avoid statistical overadjustment. In contrast, sex was not included in the CRQ, and CRQ did not explain variation in sex; however, sex differed significantly across clusters (*F*(4, 3,007) = 11.30, *p* < 0.001), and was thus treated as an independent covariate and controlled for. Full results are presented in Supplementary Table S5. Nonetheless, to ensure full transparency, we have included an additional version of the regression model that adjusts for education, occupation, and sex in Supplementary Tables S6–S10.

Power analyses, performed using the “pwr” package in R, calculated effect sizes based on Cohen’s *d* or *f*^2^, depending on the statistical test. *Ad hoc* power analysis (Supplementary Table S4) showed high power for detecting main ANOVA effects (effect size = 0.0423, power = 0.999) and several *post hoc* comparisons (e.g., A-B: 0.441, power = 0.999; A-C: 0.281, power = 0.856). However, other comparisons, such as B-D and C-E, had low power (≤0.11), increasing the risk of Type II error. In the LMM, main effects of time and cluster were well powered (power = 0.999), but the interaction term showed limited power (effect size = 0.0006, power = 0.36). These limitations are further discussed in the manuscript.

## Results

### Relationship between healthy habits and cognitive performance

The data used for the clustering analysis came from Phase 1 and included a total of 3,013 participants. Table 1 provides an overview of cluster distribution within the subgroup of participants (*n* = 666) who underwent the in-person assessment at both Wave 1 and Wave 2, revealing no significant differences in distribution across these time points. Additional details on group differences for each classification variable are available in Supplementary material.

**Table 1:** Cluster distribution in each phase.

Variable	Category	Healthy	Low cognitive reserve	Obesogenic	Heavy smokers	Alcohol-sleep	Differences between groups
On-line	N	1,249	908	432	300	124	
	%	41.4%	30.1%	14.3%	10%	4.1%	
In-person Wave 1 (Baseline)	N	343	281	118	75	38	
	%	40.11%	32.9%	13.8%	8.8%	4.4%	
In-person Wave 2 (Follow up)	N	273	209	95	58	31	
	%	41%	31.4%	14.3%	8.7%	4.6%	
Age		56.8	55.2	56.2	**60.1**	55.2	Tukey Contrasts *p* < 0.001*
Gender	Men	43%	53%	48%	59%	79%	*χ*^2^: 124 (8) *p* < 0.001 Cramer: 0.15 (small)
	Women	57%	47%	52%	41%	21%	
Education	Primary	0.6%	5.3%	3.4%	8%	0%	*χ*^2^: 124 (8) *p* < 0.001 Cramer: 0.16 (small)
	Secondary	16.0%	27.8%	27.1%	40%	28.9%	
	Superiors	83.4%	66.9%	69.5%	52%	71.1%	
Occupation	Without paid work or domestic work	1.5%	4.6%	4.3%	4.1%	0%	*χ*^2^: 49.9 (16) *p* < 0.001 Cramer: 0.10 (small)
	Qualified manual work	4.7%	13.6%	7.7%	16.4%	18.9%	
	Qualified non-manual work	15.6%	24.6%	23.9%	23.3%	18.9%	
	Profession that requires university training	32.4%	28.9%	29.1%	23.3%	29.7%	
	Profession that requires university training with people in charge	45.7	28.2%	35.0%	32.9%	32.4%	

In bold, the Heavy smokers group was significantly older than the other groups.

The mean interval between Wave 1 and Wave 2 assessments was 846.8 days (SD = 142.2). A linear regression analysis showed that this time interval was not significantly associated with observed changes in outcomes (*β* = −5.28e−06, *p* = 0.947).

Significant socio-demographic differences emerged across the five identified lifestyle clusters (Table 1). The Healthy cluster was the most frequent in all data collection waves (41.4% online; 40.1 and 41% in Waves 1 and 2, respectively) and included the highest proportion of participants with tertiary education (83.4%) and professional occupations requiring university-level training (78.1%). The Heavy Smokers cluster comprised the oldest participants (mean age = 60.1 years, *p* < 0.001), had a predominantly male composition (59%), and included the lowest proportion of individuals with higher education (52%). The Alcohol-Sleep cluster displayed the most marked gender imbalance, with 79% men, and fewer participants engaged in high-level professional roles. The Low Cognitive Reserve and Obesogenic clusters had intermediate age profiles but were more likely to include participants with primary or secondary education and with occupations involving manual or lower-qualified non-manual work. All differences in age, gender, education, and occupation were statistically significant (*p* < 0.001), with small effect sizes (Cramer’s V ranging from 0.10 to 0.16), suggesting meaningful yet modest socio-demographic stratification across clusters.

A summary of composite and domain-specific cognitive scores at baseline and follow-up for each cluster is provided in Supplementary Table S1. Supplementary Table S2 includes detailed mean scores of each individual cognitive test across time points and clusters, illustrating the specific cognitive profiles associated with each group.

**Figure 4:**
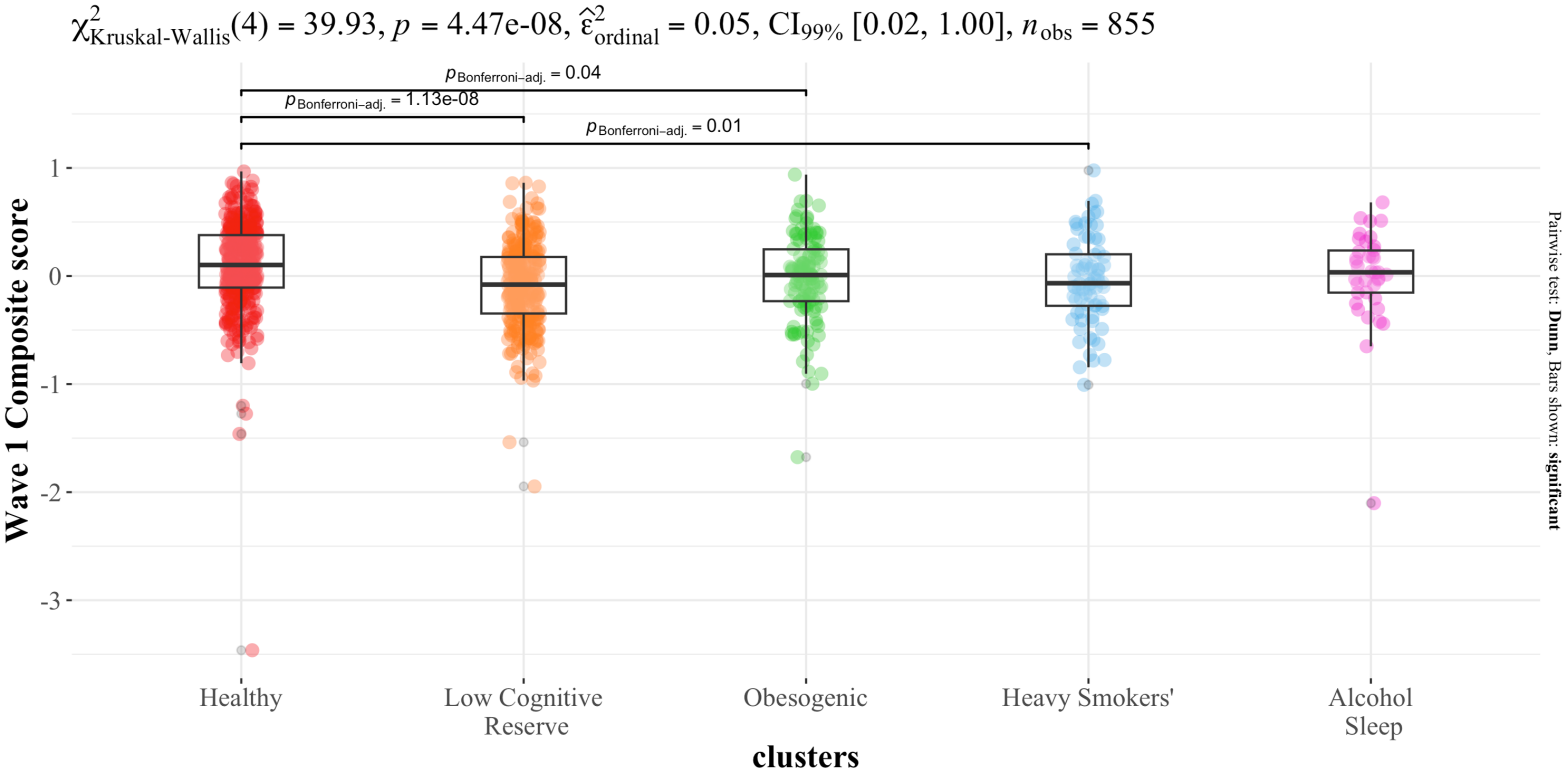
Distributions of neuropsychological assessment composite score at Wave 1 across clusters (plot obtained with the ggplot2 package, function ggbetweenstats).

As shown in Figure 4, the Kruskal-Wallis test revealed significant overall differences in Wave 1 composite scores across clusters. *Post hoc* pairwise comparisons indicated that the “Healthy” cluster scored significantly higher than the “Low Cognitive Reserve” (*p* < 0.001), “Obesogenic” (*p* < 0.05), and “Heavy Smokers” (*p* = 0.01) clusters.

To account for potential confounding of sex, we conducted a complementary linear regression analysis using ranked composite scores. This adjusted model confirmed significant differences between the “Healthy” and “Low Cognitive Reserve” (*β* = −98.33, *p* < 0.001), “Obesogenic” (*β* = −68.19, *p* = 0.009) and “Heavy Smokers” (*β* = −89.47, *p* = 0.004) clusters, while “Alcohol-Sleep” profile did not differ significantly from “Healthy” cluster (*β* = −40.77, *p* = 0.333). Sex was not significantly associated with cognitive scores (*β* = 14.52, *p* = 0.383).

Given that education and occupation are partially embedded within the CRQ scores used to form the clusters, these variables were not included in the initial models to avoid over-adjustment. However, to formally explore whether the observed cluster differences remained after controlling for residual variation in education and occupation, we conducted an extended regression including these variables. After this adjustment, the effect sizes for cluster differences were notably attenuated. The “Low Cognitive Reserve” cluster still scored significantly lower than the “Healthy” cluster (*β* = −75.05, *p* < 0.001), though with a reduced effect size (~24% smaller). Differences for the “Obesogenic” cluster were marginal (*β* = −50.47, *p* = 0.050), while the “Heavy Smokers” cluster difference was no longer statistically significant (*β* = −55.85, *p* = 0.077). The “Alcohol-Sleep” cluster remained non-significant (*β* = −24.46, *p* = 0.558).

Crucially, education emerged as a strong independent predictor of cognitive scores (*β* = 95.88, *p* < 0.001), consistent with its role in cognitive reserve, while occupation did not reach significance (*p* = 0.571) (see Supplementary Table S6).

**Figure 5:**
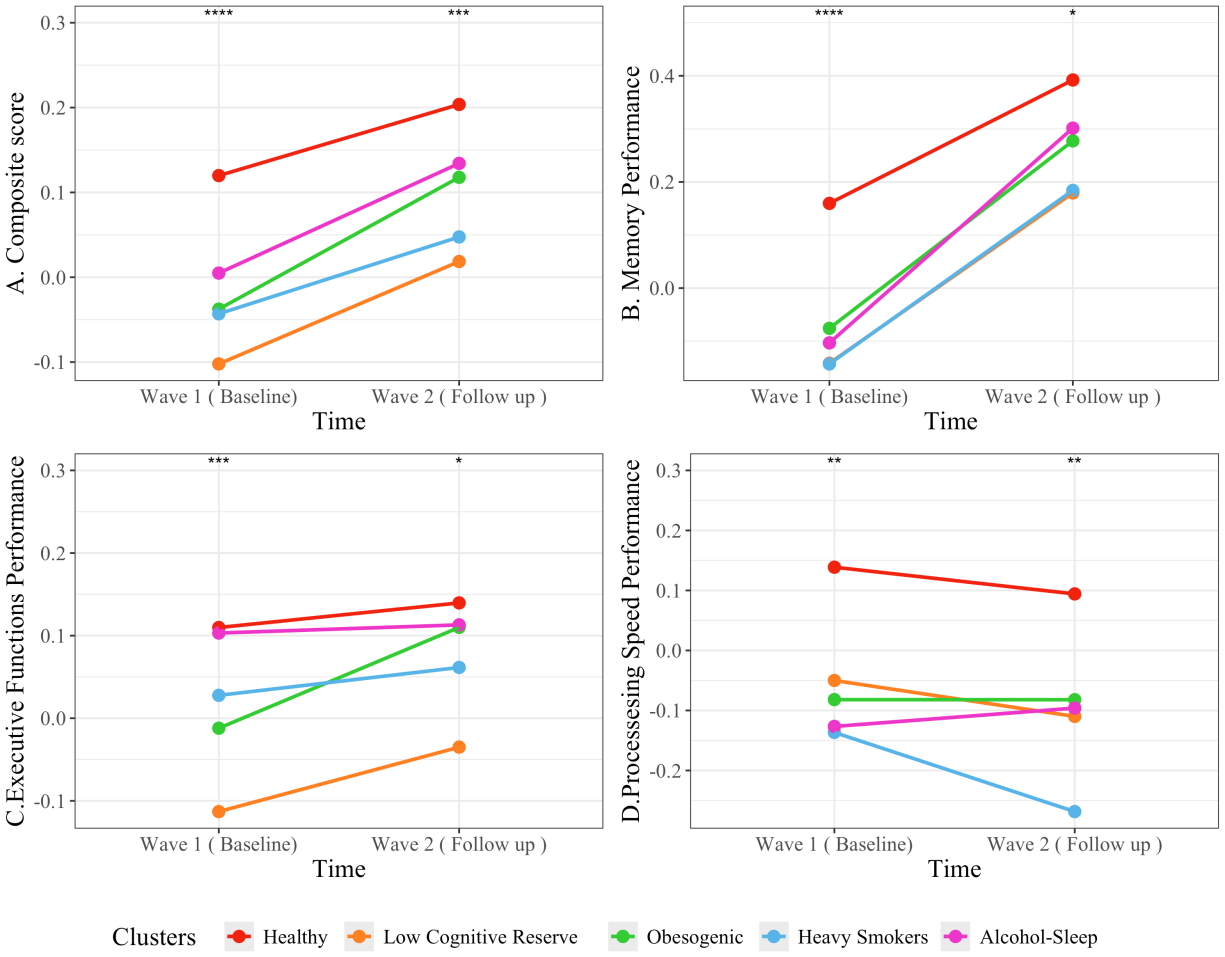
Changes from Wave 1 (Baseline) to Wave 2 (Follow-up) in **(A)** composite score, **(B)** memory, **(C)** executive functions, and **(D)** processing speed across five clusters: Healthy, Low Cognitive Reserve, Obesogenic, Heavy Smokers, and Alcohol-Sleep. Significant differences between time points are indicated by asterisks for all clusters (ns = not significant, *p* > 0.05, *p ≤ 0.05, **p ≤ 0.01, ***p ≤ 0.001, **** *p* ≤ 0.0001).

Linear mixed-effects models were used to examine changes in neuropsychological performance (NP)—including memory, executive function, and processing speed—across two time points and among clusters (Figure 5). The model for the cognitive composite score revealed a significant main effect of time, indicating improved performance from Wave 1 to Wave 2 (Estimate = 0.0833, SE = 0.0174, *t*(683) = 4.79, *p* < 0.001). Significant between-cluster differences were also observed. Participants in the “Low cognitive reserve,” “Obesogenic” clusters (*p* < 0.01) and “Heavy Smokers” cluster (*p* = 0.01) showed significantly lower cognitive scores compared to “Healthy” cluster. In contrast, the “Alcohol-Sleep” cluster did not significantly differ from the “Healthy” cluster (*p* = 0.191). These effects remained significant after adjusting for sex, which was not a significant predictor of cognitive performance (*p* > 0.05). However, the interaction terms between time and clusters were not significant, indicating that the rate of change in cognition over time did not significantly differ between clusters (all *p* > 0.05).

To explore whether residual differences in education or occupation contributed to these results, we fitted a secondary model (Model 2; Supplementary Table S7) that included these variables. In this extended model, education emerged as a strong independent predictor of cognitive performance (Estimate = 0.196, SE = 0.038, *t*(844) = 5.14, *p* < 0.001), whereas occupation remained non-significant (*p* = 0.610). After adding these covariates, the magnitude of the differences between clusters was notably reduced. The estimated difference between the “Low Cognitive Reserve” and “Healthy” clusters decreased from −0.222 to −0.175 but remained highly significant (*p* < 0.001). The difference between the “Obesogenic” and “Healthy” clusters was also attenuated to −0.123 and, although still statistically significant, showed a higher *p*-value (*p* = 0.021). In contrast, the previously observed difference for the “Heavy Smokers” cluster was further diminished and no longer reached statistical significance (Estimate = −0.095, *p* = 0.145). The “Alcohol-Sleep” cluster continued to show no significant difference compared to the “Healthy” group (*p* = 0.331).

For memory performance (z-scored), there was a significant improvement over time (Estimate = 0.227, SE = 0.0377, *t*(691.01) = 6.02, *p* < 0.001), indicating higher scores at follow-up. Compared to the “Healthy” cluster, participants in the “Low cognitive reserve” (*p* < 0.001), “Obesogenic” (*p* = 0.0085) and “Heavy Smokers” (*p* = 0.009) clusters had significantly lower memory scores, while no significant differences were observed for the “Alcohol-Sleep” cluster (*p* = 0.213). Interaction terms between time and clusters were not statistically significant (all *p* > 0.05), indicating similar memory improvements across groups. These effects were observed after controlling for sex that was significantly associated with female sex (*p* < 0.001). To further explore if differences in education or occupation accounted for these associations, an additional model (Model 2; Supplementary Table S8) was fitted including these variables. Education emerged as a strong independent predictor of memory scores (Estimate = 0.320, SE = 0.058, *t*(833.12) = 5.51, *p* < 0.001), whereas occupation remained non-significant (*p* = 0.294). After including education and occupation, the magnitude of the differences between clusters was attenuated. The estimated difference between the “Low Cognitive Reserve” and “Healthy” clusters decreased from −0.274 to −0.222, although it remained highly significant (*p* < 0.001). The difference for the “Obesogenic” cluster was also reduced to −0.185 and retained statistical significance with a higher *p*-value (*p* = 0.027). In contrast, the difference for the “Heavy Smokers” cluster diminished further and was no longer statistically significant (Estimate = −0.163, *p* = 0.112). The “Alcohol-Sleep” cluster continued to show no significant difference compared to the “Healthy” group (*p* = 0.269).

For executive functions, there was a non-significant trend toward improvement over time (Estimate = 0.04, SE = 0.0271, *t*(709) = 1.49, *p* = 0.138). The “Low cognitive reserve” (B; *p* < 0.001) and the “Obesogenic” cluster (*p* = 0.045) showed significantly lower executive function scores than the “Healthy” cluster, while differences with the “Heavy Smokers” (*p* = 0.20) and “Alcohol-Sleep” (*p* = 0.692) clusters were not significant. No significant time-by-cluster interactions were found (all *p* > 0.05). These results were obtained while controlling for sex, significantly associated male sex (*p* = 0.015). An additional model (Model 2; Supplementary Table S9) was fitted to explore whether residual differences in education or occupation could account for these associations. Education emerged as a significant independent predictor of executive function scores (Estimate = 0.154, SE = 0.044, *t*(854) = 3.46, *p* < 0.001), whereas occupation did not show a significant association (*p* = 0.506). Including these variables attenuated the differences between clusters. The estimated difference for the “Low Cognitive Reserve” cluster relative to the “Healthy” cluster decreased from −0.233 to −0.193 but remained highly significant (*p* < 0.001). In contrast, the difference for the “Obesogenic” cluster was reduced to −0.094 and no longer reached statistical significance (*p* = 0.138). Differences for the “Heavy Smokers” and “Alcohol-Sleep” clusters remained non-significant.

For processing speed, there was a non-significant trend toward decline over time (Estimate = −0.0537, SE = 0.035, *t*(695) = −1.52, *p* = 0.128). The “Low cognitive reserve,” “Obesogenic,” and “Heavy Smokers” clusters showed significantly lower processing speed scores than the “Healthy” cluster (all *p* < 0.05). The “Alcohol-Sleep” cluster did not differ significantly from the “Healthy” cluster (*p* = 0.147). No significant time-by-cluster interactions were found (all *p* > 0.05). Adjusting for sex showed that female participants had higher processing speed scores (*p* = 0.0026). In an extended model (Model 2; Supplementary Table S10) that additionally included education and occupation, education emerged as a significant positive predictor of processing speed (Estimate = 0.236, SE = 0.062, *t*(845) = 3.79, *p* < 0.001), whereas occupation remained non-significant (*p* = 0.405). After adjusting for these factors, the differences between clusters were slightly amplified for most comparisons. The estimated difference for the “Low Cognitive Reserve” cluster relative to the “Healthy” cluster became −0.189 and remained significant (*p* = 0.005). The “Obesogenic” cluster also showed a strengthened association (Estimate = −0.221, *p* = 0.013), and the “Heavy Smokers” cluster exhibited an even larger difference (Estimate = −0.275, *p* = 0.009). Interestingly, the difference for the “Alcohol-Sleep” cluster approached significance after adjustment (Estimate = −0.265, *p* = 0.051).

## Discussion

The primary objective of this study was to analyze how combinations of lifestyle behaviors cluster together and relate to cognitive performance at a specific point in time, as well as to begin exploring how these relationships evolve longitudinally. Using data from a cohort of healthy middle-aged adults, the study aimed to examine variations in cognitive trajectories across five stable lifestyle clusters. Identifying these profiles provides valuable insights into how specific combinations of lifestyle factors are associated with different levels of cognitive functioning.

Subjects classified under the “Healthy” profile were individuals who adhered to a Mediterranean diet, engaged in regular physical exercise, had strong social networks, reported good sleep perception, high scores in purpose in life, and actively sought cognitive stimulation (Roca-Ventura et al., 2024). This profile, when compared to other profiles, had the healthiest behaviors, and displayed the highest levels of cognitive performance. In contrast, clusters characterized by riskier habits, such as “Obesogenic” and “Heavy Smokers,” displayed lower cognitive performance compared to the “Healthy” profile.

The “Low cognitive reserve” profile included individuals with minimal engagement in cognitively stimulating activities, limited social interactions, and poor planning of vital life aspects. This group also had significantly lower levels of education compared to the “Healthy” profile. Consistent with these characteristics, individuals in the low reserve group demonstrated reduced cognitive performance across all domains. These results underscore the significance of cognitive activities and education, modifiable factors that influence cognitive performance from early life stages (Livingston et al., 2020). Education has well-established protective effects and is consistently linked to better cognitive outcomes (Thow et al., 2017; Wilson et al., 2019). Although education was partially embedded in the clustering process through the CRQ, our additional analyses demonstrated that it remained an independent predictor of cognitive performance, partially attenuating but not eliminating the differences between clusters. This underscores the closely related yet distinct contributions of formal education and broader lifestyle-cognitive reserve profiles to cognitive outcomes in this cohort. Importantly, this highlights that education’s influence extends beyond its early-life effects, fostering engagement in intellectually enriching activities that can help maintain or enhance specific cognitive abilities, particularly in older adults. While the impact of education is often most pronounced in early life, cognitive stimulation in later life can also play a crucial role in preserving cognitive health (Ruppert et al., 2024). Moreover, benefits of education are not solely determined by the number of years spent in school. When occupational complexity is considered, the direct effect of education on dementia risk may diminish. This indicates that the long-term cognitive advantages of education depend on its application, particularly through mentally stimulating work. Indeed, individuals with more education who did not pursue cognitively demanding careers were not at a lower risk of dementia compared to those with less education but more complex occupational roles (see also Karp et al., 2004). These findings support the notion that cognitive reserve—and its protective effects on cognition—is best understood within a broader life course model. It is not solely built through early-life education, but through the continued translation of that education into intellectually engaging life experiences. This underscores the importance of promoting not only educational access, but also lifelong opportunities for cognitive stimulation as a strategy to support cognitive health and reduce the risk of dementia.

It is also noteworthy in this cluster the co-occurrence of cognitive activity and poor socialization, where poor socialization may exacerbate the impact of limited cognitive activity. These results align with previous literature suggesting that cognitive reserve moderates the association between social isolation and cognitive outcomes in later life (Evans et al., 2018). Based on the cognitive reserve theory, social integration would provide mental stimulation through complex communication and interaction with others. Furthermore, the results also highlight the potential role of vital plan in promoting cognitive resilience during middle age (Boyle et al., 2010; Abellaneda-Pérez et al., 2023). These three variables—cognitive activity, socialization, and vital plan—appear interconnected, each moderating the other, and carry important implications for interventions that may target social isolation, vital plan, and cognitive activity simultaneously, thereby adopting a holistic approach to improve cognitive function.

Conversely, the “Alcohol-Sleep” cluster, which also showed poor vital plan and socialization but have higher cognitive activities than “Low Cognitive reserve” and more level of education than the other risky profiles, did not significantly differ from the “Healthy” cluster in cognitive performance. This group also had higher levels of stress and greater prevalence of psychiatric diseases such as depression and anxiety. This finding aligns with studies such as Yu et al. (2020), which found that better cognition scores (super-cognition) were associated with busier, more socially-isolated and stressful midlife—characteristics that resemble our “Alcohol-Sleep” group. These findings suggest that although the ‘Alcohol-Sleep’ cluster exhibited higher prevalence of stress, poor sleep perception, and mental health issues, they also had higher levels of education and cognitive activity than other high-risk clusters. This may have conferred a cognitive protective effect, buffering the impact of negative lifestyle factors. This result is consistent with previous research indicating that cognitive reserve, particularly in the form of lifelong cognitive engagement and education, can moderate the impact of adverse conditions on cognitive outcomes (Stern, 2012; Stern et al., 2019; Kremen et al., 2019). On the other hand, lack of consistent cognitive engagement throughout life, such as learning a new language or musical instruments, could impact cognitive function and increase the risk of dementia (Ihle et al., 2015; Lee et al., 2018; Leggieri et al., 2019; Bae et al., 2020). A more extensive longitudinal study is needed to clarify the trajectory of these cognitive differences. This approach could provide more precise insights into whether early-life cognitive activity is associated with better cognitive performance in middle age, while other risk factors may start to impact cognitive function in later life.

Indeed, when we looked at longitudinal changes we found a moderate increase over time in cognitive performance. The observed improvement across all lifestyle clusters, including those with riskier profiles, warrants careful interpretation. Given the characteristics of the cohort—cognitively healthy middle-aged adults—and the relatively short follow-up period (approximately 2 years), an overall upward trend in neuropsychological test scores is not totally surprising. Previous research has shown that cognitively healthy individuals in midlife may improve performance when exposed repeated cognitive assessments over short intervals, possibly due to practice effects, familiarity and test-taking strategies (Bartels et al., 2010; Kremen et al., 2022).

These effects could be particularly evident when participants present relatively high educational attainment and socioeconomic status (Cabeza et al., 2018; Stern, 2012; Stern et al., 2019; Stern et al., 2020), as in the case of BBHI’s participants (Cattaneo et al., 2020). These findings suggest that lifestyle-related cognitive differences may become more pronounced over longer follow-up periods or with the inclusion of older populations approaching the threshold of age-related cognitive decline.

However, our findings highlight the beneficial impact of engaging in multiple health behaviors from middle-aged to older adults on cognitive performance. Jia et al. (2023) proposed a framework for classification based on the number of adhered healthy lifestyle factors, defining an unfavorable group for those following 0–1 factor, an average group for 2–3 factors, and a favorable group for 4–6 factors. This classification demonstrated that the unfavorable group was linked to greater memory deterioration compared to the average and favorable groups, even among individuals carrying the APOE ε4 allele. In our investigation, the “Healthy” group, similar to Jia et al.’s favorable group, showed superior cognitive performance compared to clusters engaged to more than one unhealthy behavior, which likely correspond to Jia et al.’s unfavorable or average groups. Similarly, Dhana et al. (2024) explored the association between lifestyle choices and cognitive function in older adults, revealing that adhering to a healthy lifestyle could potentially provide cognitive reserve to maintain cognitive abilities despite the presence of brain neuropathologies. Nonetheless, these models assume that behaviors are interchangeability, whereas our analysis has revealed profiles characterized by the coexistence of healthy and unhealthy behaviors, akin to Jia et al. (2023) average group, with distinct impacts on cognitive performance. This suggests that specific combinations of modifiable factors may be more influential on cognitive maintenance, and that adhering to a higher number of healthy behaviors positively impacts cognitive performance. Multiple studies have found associations between cognitive activities such years of schooling, engage in training courses, play music instruments or professional occupation and cognitive function moderate or attenuate the association between brain pathology and the clinical outcomes (Song et al., 2022), however this study also highlights the importance of other factors such as vital plan an socialization in this moderation.

Recent years have shown a rise in studies examining lifestyle factors like nutrition, physical exercise, tobacco, and alcohol consumption. However, certain key lifestyles have been overlooked as essential components of modifiable health factors. Liao et al. (2022) included social activity in their clustering analyses of the association between health-related behaviors and episodic memory. The results revealed the importance of engaging in social and physical activities for the preservation of episodic memory in old age. Our study is the most comprehensive to date, analyzing traditional lifestyle factors along with less conventional ones, creating unique profiles of co-occurring behaviors that have not been previously studied (Livingston et al., 2020; Abellaneda-Pérez et al., 2023). The aggregation of these factors into unique lifestyle profiles—and their association with cognitive outcomes—has not been previously explored, underscoring the novelty and value of this study. Public health experts, legislators, and healthcare providers can use this knowledge to better target programs and encourage positive lifestyle changes, leading to more effective and efficient use of resources and interventions, improving people’s quality of life and health outcomes.

The effectiveness of multimodal therapies, which address multiple risk factors simultaneously, is often higher than single-domain interventions, but results have been inconsistent. A meta-analysis by Meng found moderate evidence indicating that multidomain interventions can improve cognitive composite scores but no moderate- or high-certainty evidence that these interventions enhance global cognition (Meng et al., 2022). However, these interventions are more promising when targeting at-risk populations (Kivipelto et al., 2018), as seen in trials like the Finnish Geriatric Intervention Study (FINGER, Kivipelto et al., 2013; Ngandu et al., 2015), the Prevention of Dementia by Intensive Vascular Care (PreDiva), and Multidomain Alzheimer Preventive Trial (MAPT, Vellas et al., 2014) which showed cognitive benefits in higher-risk subgroups. These results, along with our research, point to the importance of multimodal approach for modifying lifestyles habits and improving cognitive reserve. However, it is essential to acknowledge that personalized and profile-specific interventions are necessary considering that lifestyle patterns have been linked to distinct cognitive trajectories and lifestyle habits tend to cluster differently. Considering the persistent nature of these habits, it is ineffective to use universal techniques to encourage sustainable behavior change. Instead, interventions should be tailored to target individual lifestyle profiles and address the particular obstacles and problems that are distinct to each profile. It is more reasonable to implement and sustain healthy lifestyle changes over time when programs are individualized and provide continuous support.

The use of machine learning algorithms like KmL3D enables the complex analysis and identification of joint trajectories in longitudinal data into unique lifestyle profiles, that have proven to be not only adequate and reliable but also highly valuable for revealing insights that would be challenging to obtain using other approaches. This advanced approach has allowed the identification of patterns and relationships within the data that would remain hidden in simpler analyses, offering deeper understanding of how lifestyle choices influence cognitive aging over time. By leveraging KmL3D, the study can capture the dynamic and multifaceted nature of lifestyle behaviors and how they are differently associated with cognition. By capturing the complexity of real-world behaviors and their impact on cognitive outcomes, this methodology enhances our ability to predict, prevent, and manage cognitive decline more effectively. This kind of analysis could lead to more accurate and personalized predictions and interventions that could significantly improve public health strategies and individualized care.

Taken together our findings suggest that lifestyle clustering based on longitudinal data may be a valuable strategy for identifying middle-aged adults at increased risk of future cognitive decline. Personalized interventions targeting multiple modifiable risk factors simultaneously—especially those addressing cognitive engagement, socialization, and goal-setting—may enhance resilience to age-related cognitive deterioration. By focusing on the specific lifestyle profiles uncovered in this study, tailored interventions could more effectively strengthen cognitive reserve and delay the onset of clinically relevant decline. Given that lifestyle patterns tend to cluster and remain stable over time, universal approaches to encourage behavior change may be less effective. Instead, interventions tailored to individual profiles that account for unique challenges and barriers are likely to be more sustainable and impactful. Such tailored programs, offering continuous support, may facilitate the long-term adoption of healthy behaviors and thereby help preserve cognitive function. Future studies should explore these profiles across a longer time span and in more diverse populations to test the generalizability of the current findings.

## Limitations

In conclusion, this study demonstrates the feasibility and relevance of using unsupervised clustering on lifestyle trajectories to identify cognitive profiles in healthy adults. However, some limitations must be acknowledged. First, the reliance on self-reported data may introduce bias. Second, sample characteristics in terms of age-range and health status, and the relatively short follow-up period, could have generated practice effects possibly responsible of the small cognitive improvements observed, and preventing to observe meaningful cognitive changes over time and differences between clusters. Third, the sample includes predominantly highly educated and health-conscious individuals, limiting generalizability. Fourth, some of the statistical analyses were underpowered—particularly the interaction term in the multilevel model (MLM) and several *post hoc* comparisons in the ANOVA (see Supplementary material for details). This limited statistical power increases the risk of Type II errors, meaning that potentially meaningful effects may not have reached statistical significance. Therefore, results derived from these specific analyses should be interpreted with caution. Future studies should aim for larger and more balanced samples, as well as longer follow-up periods, to confirm and extend these findings.

## Data Availability

The raw data supporting the conclusions of this article will be made available by the authors without undue reservation.
