# Supplementary material for: Clustering on longitudinal lifestyle trajectories and their impact on cognitive performance
Source: Front Psychol. 2025 Jul 25;16:1510971. doi: 10.3389/fpsyg.2025.1510971 (PMC12333451; doi:10.3389/fpsyg.2025.1510971)
Supplement: Supplementary file 1 [file Data_Sheet_1.pdf]

1.Measured Variables

1.1 Cognitive Activity

Cognitive activity was measured using the Cognitive Reserve Questionnaire (CRQ; (Rami et al., 2011), a validated tool for the Spanish population, applicable to both healthy individuals and those in the early stages of Alzheimer's disease. This brief instrument includes eight items covering cognitively stimulating experiences, such as academic background of both participants and their parents, professional roles, music education, and language skills. Scores range from 0 to 25, with higher scores reflecting greater cognitive reserve.

1.2 Physical Activity

Participants self-reported their activity levels using the International Physical Activity Questionnaire (IPAQ ; (Craig et al., 2003). This tool captures the frequency and duration of different types of physical activity (e.g., leisure, transportation, and occupational), distinguishing between moderate, vigorous, walking activity, and inactivity over the past seven days. Examples and physiological cues are provided to aid recall. Activities were then translated into Metabolic Equivalent of Task (MET) scores and categorized by intensity following IPAQ analysis guidelines.

1.3 Social Engagement

Social connectedness was evaluated with the Lubben Social Network Scale, revised version (LSNS; (Lubben, 1988), which identifies potential social isolation in older adults. The instrument assesses perceived social support from family, friends, and neighbors based on the number, closeness, and frequency of contacts. Scores range from 0 to 60, with higher scores indicating stronger social support.

1.4 Dietary Habits

Adherence to the Mediterranean diet was measured using the MEDAS-14 questionnaire (Schröder et al., 2011), which consists of 14 items—12 addressing food frequency and 2 relating to specific dietary habits typical of the Mediterranean lifestyle. Each item is scored 0 or 1, with total scores ranging from 0 to 14, indicating the level of adherence to this dietary pattern.

1.5 Purpose and Personal Growth

To assess psychological well-being related to life purpose and personal growth, two subscales from Ryff's Psychological Well-being Scale were administered. Each subscale includes seven items rated on a 6-point Likert scale (from strong disagreement to strong agreement). Combined, they yield a score ranging from 14 to 84, with higher scores denoting greater well-being in these areas (Ryff, 1995).

1.6 Sleep Quality

Sleep disturbances were assessed using the Jenkins Sleep Scale (JSS; (Jenkins et al., 1988), a short self-report tool consisting of four questions regarding sleep issues experienced over the past month. It includes items about sleep onset, maintenance, nighttime awakenings, and daytime fatigue. Responses are rated on a 0–5 Likert scale, with total scores ranging from 0 to 20. Higher scores reflect more frequent or severe sleep difficulties.

1.7 Obesity

Body Mass Index (BMI) was used as an indicator of obesity. Although more precise measures such as waist circumference or body fat percentage exist, BMI remains a widely accepted standard. It was calculated by dividing weight in kilograms by the square of height in meters (kg/m^2), using data collected at each assessment.

1.8 Alcohol Intake

Alcohol use was evaluated using the Alcohol Use Disorders Identification Test (AUDIT; (Conigrave et al., 1995), a globally recognized screening tool developed by the World Health Organization. It includes questions on consumption patterns, potential consequences of drinking, and signs of dependency. Each question is rated from 0 to 4, producing a total score between 0 and 40. Scores of 8 or higher suggest hazardous drinking behavior.

1.9 Tobacco Use

Lifetime tobacco exposure was calculated using the pack-year method (Duriez et al., 2014; Franklin et al., 2014; Karama et al., 2015; Bittner et al., 2021), which multiplies the number of years the individual has smoked by the average number of cigarettes smoked per day (divided by 20 to convert to packs). This measure provides a cumulative index of tobacco use across the lifespan.

Table S1 presents the mean scores for the composite cognitive measure and the main cognitive domains (memory, executive functions, and processing speed) at both baseline and follow-up, stratified by the five data-driven clusters: A) Healthy, B) Low Cognitive Reserve, C) Obesogenic, D) Heavy Smokers, and E) Alcohol-Sleep. This overview provides a simplified summary of cognitive trajectories across clusters.

Table S1. Mean Composite and Domain-Specific Cognitive Scores at Baseline and Follow-Up, by Cluster

		CLUSTERS				
	**Timepoint**	**A. Healthy**	**B. Low Cognitive Reserve**	**C. Obesogenic**	**D. Heavy Smokers**	**E. Alcohol Sleep**
**Composite Score**	Baseline	0.12	-0.10	-0.04	-0.04	0.00
	Follow-up	0.20	0.02	0.12	0.05	0.13
**Memory Score**	Baseline	0.16	-0.14	-0.08	-0.14	-0.10
	Follow-up	0.39	0.18	0.28	0.18	0.30
**Executive Functions**	Baseline	0.11	-0.11	-0.01	0.03	0.10
	Follow-up	0.14	-0.03	0.11	0.06	0.11
**Processing Speed**	Baseline	0.14	-0.05	-0.08	-0.14	-0.13
	Follow-up	0.09	-0.11	-0.08	-0.27	-0.10

Table S2 displays the mean scores on each cognitive test used in the assessment battery, shown at both baseline and follow-up for each of the five clusters. These measures span domains such as associative and episodic memory, executive function, processing speed, attention, and intelligence. This detailed breakdown complements the summary information in Table 1 and helps illustrate specific cognitive profiles associated with each cluster.

Table S2. Mean Scores on Individual Cognitive Tests at Baseline and Follow-Up, by Cluster

			CLUSTERS				
**Cognitive Function**	**Test**	**Timepoint**	**A. Healthy**	**B. Low Cognitive Reserve**	**C. Obesogenic**	**D. Heavy Smokers**	**E. Alcohol Sleep**

**Associative memory**	**S-FNAME**	Baseline	46.25	42.53	42.55	38.98	42.85
		Follow-up	46.00	43.21	43.95	38.38	48.00
**Global cognitive status**	**MMSE**	Baseline	29.82	29.79	29.78	29.68	29.71
		Follow-up	29.87	29.83	29.85	29.82	29.81
**Working memory**	**Digit direct**	Baseline	6.22	6.01	6.14	6.31	6.08
		Follow-up	6.28	6.14	6.23	6.38	6.35
	**Digits Backward**	Baseline	5.02	4.75	4.85	5.08	4.97
		Follow-up	5.14	4.82	5.02	5.22	5.39
	**Letter number sequencing**	Baseline	5.89	5.53	5.74	5.87	5.89
		Follow-up	5.82	5.59	5.86	5.72	5.84
**Phonemic Fluency**	**Phonemic Fluency**	Baseline	19.16	17.88	18.35	18.61	19.11
		Follow-up	19.36	18.40	19.17	18.36	20.06
**Semantic Fluency**	**Semantic Fluency**	Baseline	24.49	23.66	23.66	23.91	23.66
		Follow-up	25.54	24.18	24.39	24.84	24.90
**Premorbid intelligence**	**TAP**	Baseline	27.24	26.54	27.08	27.45	26.74
		Follow-up	27.58	27.04	27.21	27.22	27.06
**General/fluid intelligence**	**Matrix Reasoning**	Baseline	20.36	19.52	20.03	19.77	20.55
		Follow-up	20.57	19.75	19.91	20.24	20.45
**Processing speed/ selective attention**	**Digit Symbol Substitution**	Baseline	79.77	77.42	76.69	74.00	77.29
		Follow-up	79.11	77.30	76.76	74.03	76.42
	**Cancelation test**	Baseline	44.70	42.63	43.35	43.31	41.32
		Follow-up	44.17	41.62	42.48	40.86	42.45
	**TMT-A**	Baseline	27.08	27.37	27.75	29.61	28.34
		Follow-up	25.24	26.24	26.59	28.41	27.29
**Cognitive flexibility**	**TMT-B**	Baseline	77.06	81.42	81.34	78.20	75.95
		Follow-up	76.06	80.08	77.60	75.66	84.42
**Episodic memory**	**RAVLT-Immediate**	Baseline	53.64	50.21	51.20	50.40	51.84
		Follow-up	58.33	55.01	56.53	55.72	57.35
		Baseline	11.80	11.02	10.91	10.75	11.32

	**RAVLT-Delayed**	Follow-up	12.86	12.05	12.35	12.34	12.55
	**RAVLT - Recognition**	Baseline	14.50	14.20	14.28	14.19	14.35
		Follow-up	14.64	14.52	14.60	14.59	14.50
**Visuo-spatial abilities**	**Block design**	Baseline	47.56	45.16	46.08	43.63	44.55
		Follow-up	47.11	45.02	47.13	44.91	46.39

Table S3 presents the mean scores and standard deviations of all variables used to define the clusters, while the corresponding statistical comparisons between clusters are shown in the Figures S1–S9 to illustrate these differences in detail.

Table S3. Mean and Standard deviation of all variables used to define the clusters

	A.Healthy	***B.Low Cognitiv Reserve***	***C.Obesogenic***	***D.Heavy Smokers***	***E.Alcohol-Sleep***
Cognitive Activity	15.41 (2.53)	10.68 (3.32)	13.23 (3.37)	13.57 (3.55)	13.28 (3.85)
Physical Exercise	3302.64 (2087.93	2414.12 (1775.59	2039.60 (1774.81	2460.64 (1749.22	2894.72 (2305.26
Socialization	40.76 (6.37)	30.88 (8.25)	35.78 (7.62)	35.34 (7.94)	34.01 (8.62)
Sleep perception	6.95 (2.35)	8.58 (3.30)	9.10 (3.83)	7.85 (2.84)	9.76 (4.14)
Nutrition	9.14 (1.45)	7.87 (1.57)	7.46 (1.60)	8.07 (1.76)	8.41 (1.91)
Vital Plan	58.55 (5.46)	48.10 (6.52)	53.69 (7.23)	50.45 (8.58)	50.06 (8.08)
Alcohol	2.27 (1.52)	2.02 (1.47)	1.89 (1.46)	2.91 (1.69)	8.98 (2.69)
Tobbaco	2.96 (5.34)	2.91 (4.69)	4.85 (7.27)	31.99 (12.32)	11.23 (9.35)
BMI	23.84 (2.73)	23.96 (2.70)	31.29 (3.49)	26.89 (4.23)	26.07 (4.16)

Kruskal–Wallis comparisons for the nine lifestyle behaviors included in the k-means clustering analysis. These comparisons were conducted without adjusting for sex as a potential confounding variable.

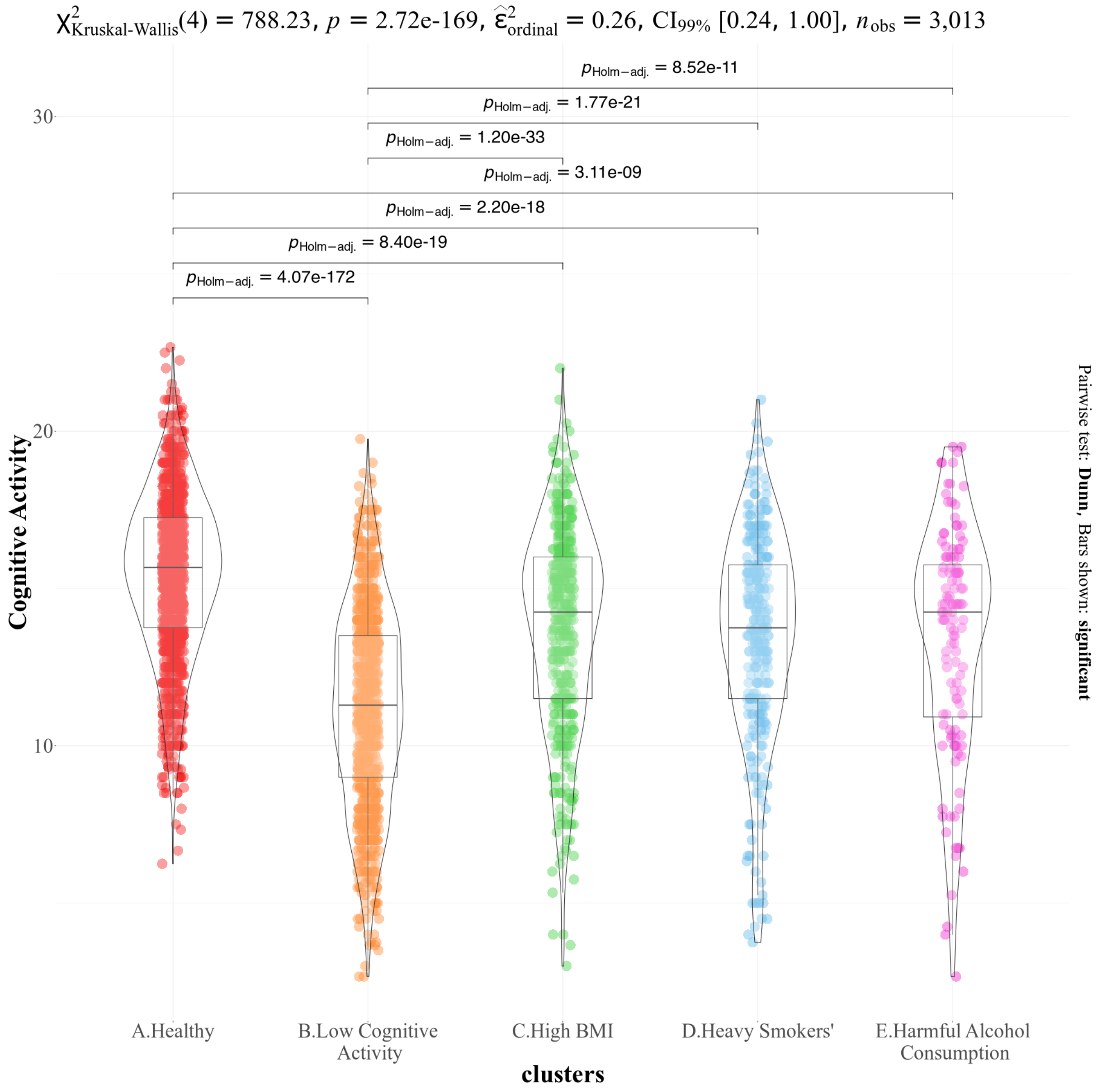

Figure S1. Comparison of Cognitive activity mean score across clusters.

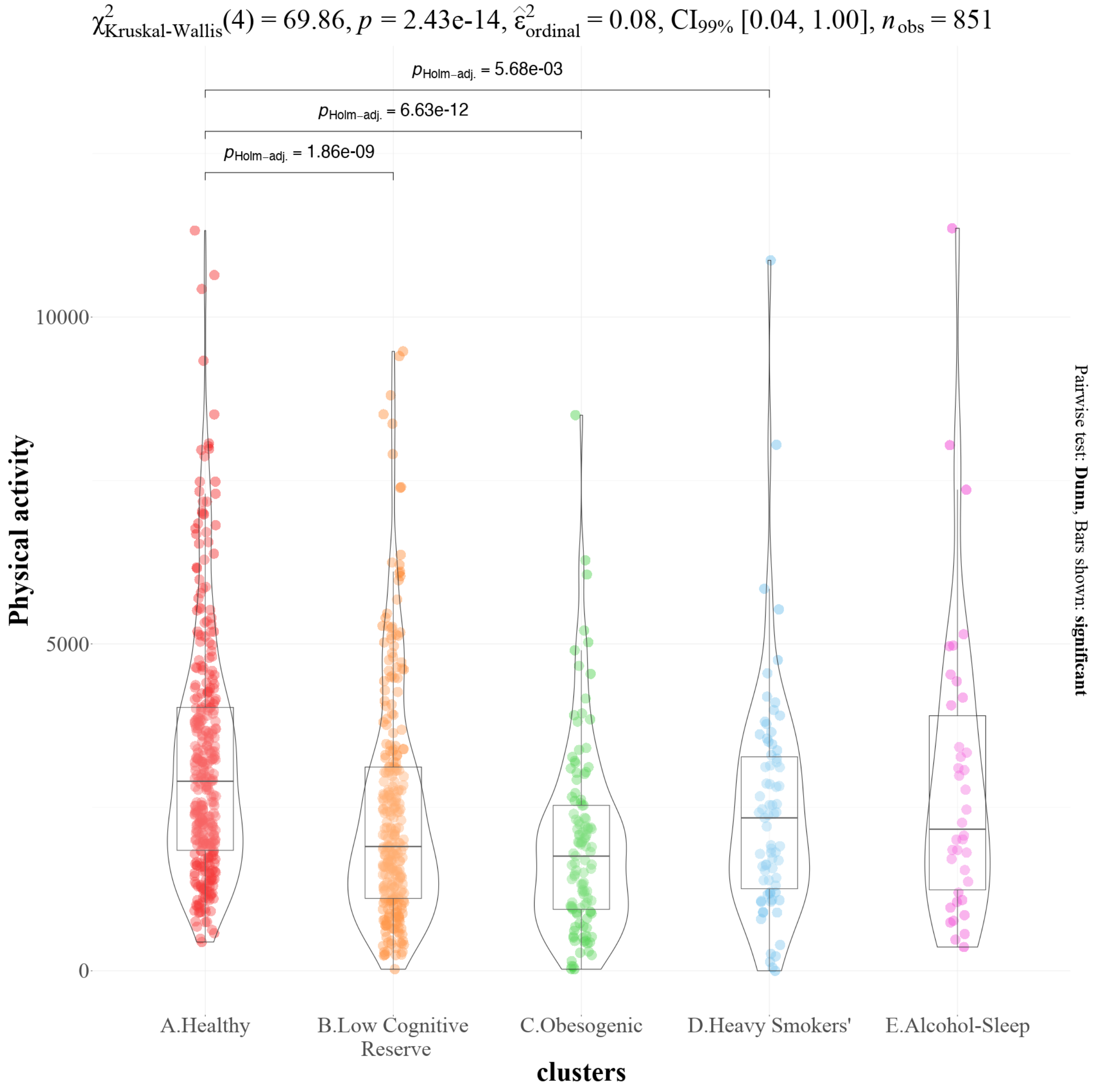

Figure S2 . Comparison of physical activity mean score across clusters.

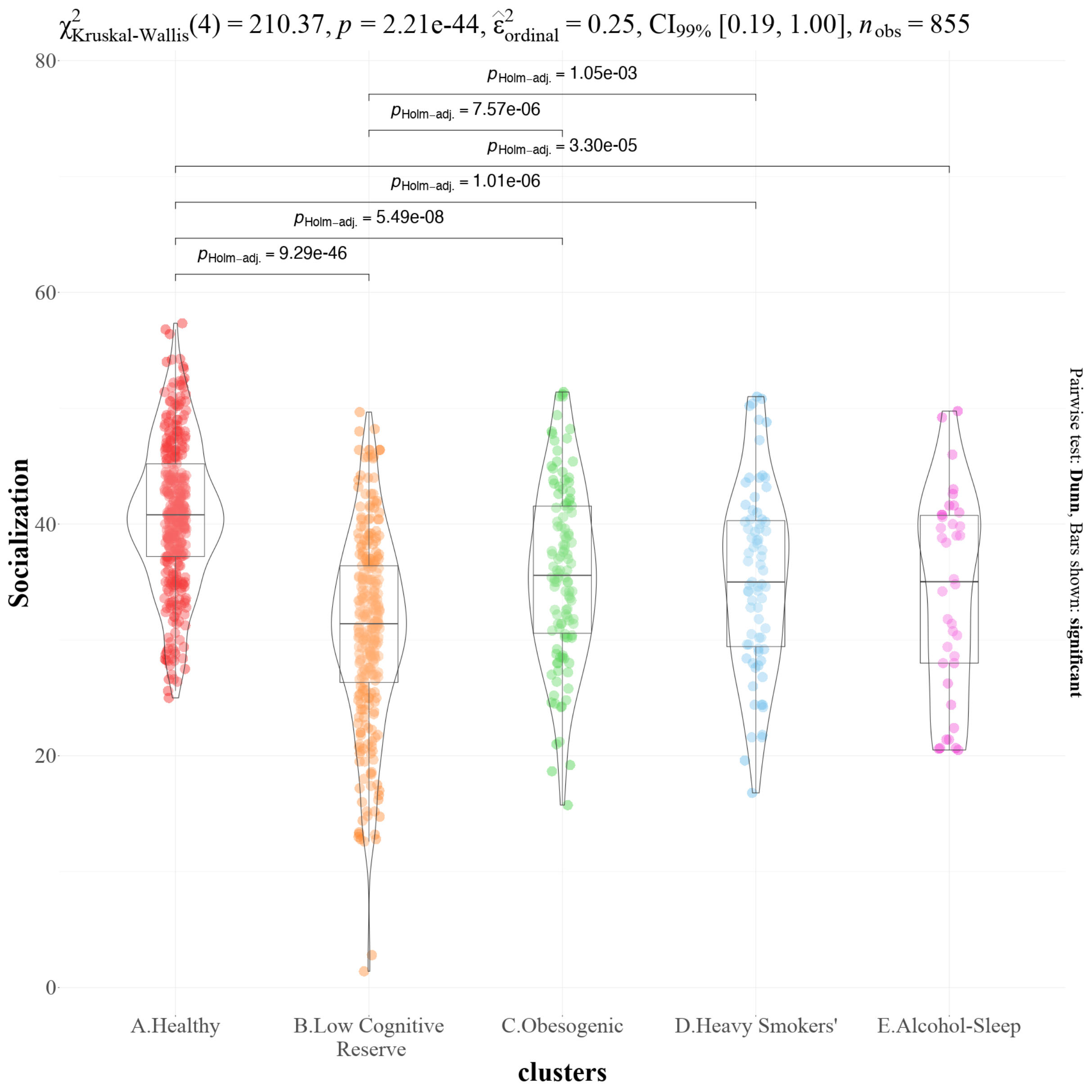

Figure S3. Comparison of socialization mean score across clusters.

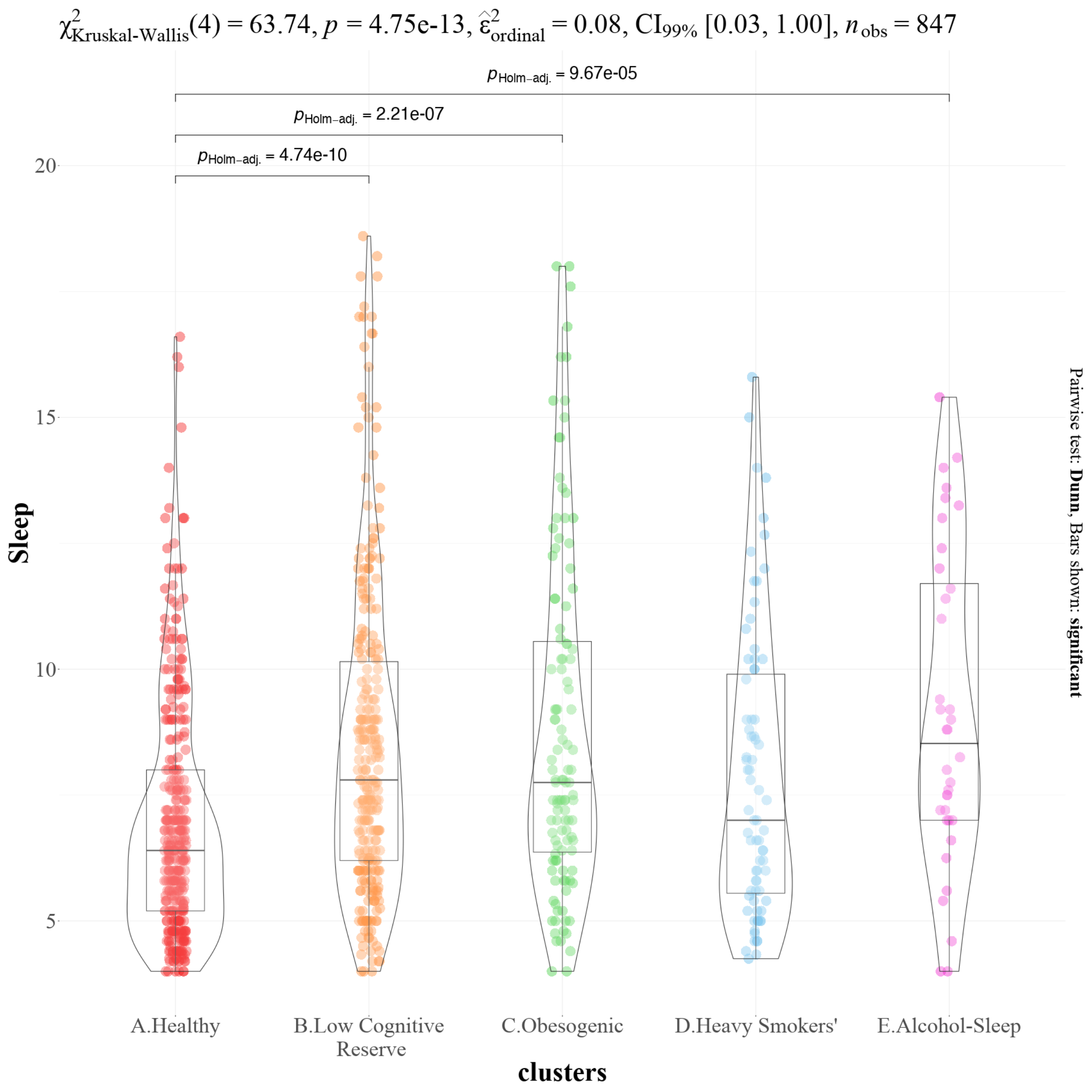

Figure S4. Comparison of Sleep mean score across clusters.

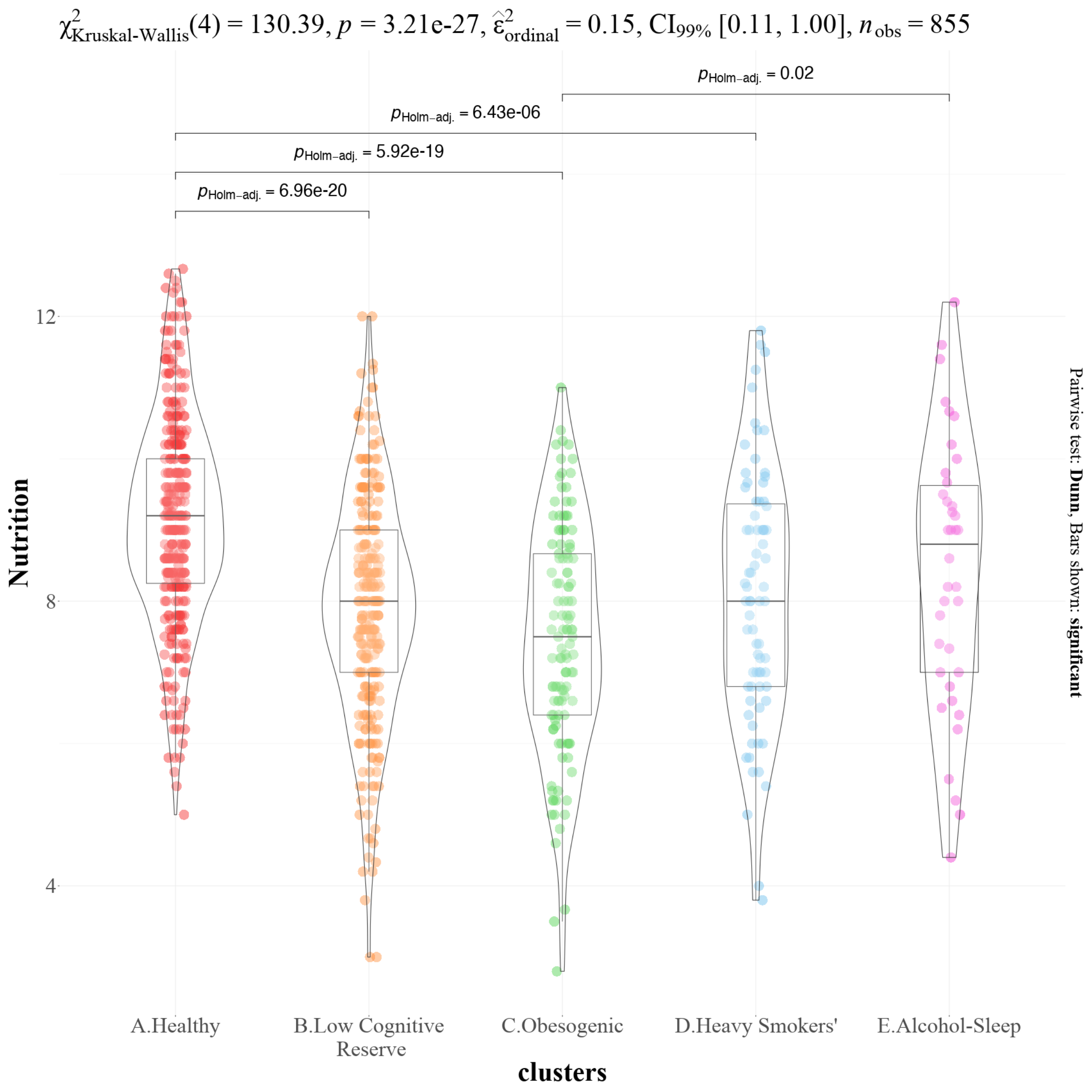

Figure S5. Comparison of Nutrition mean score across clusters.

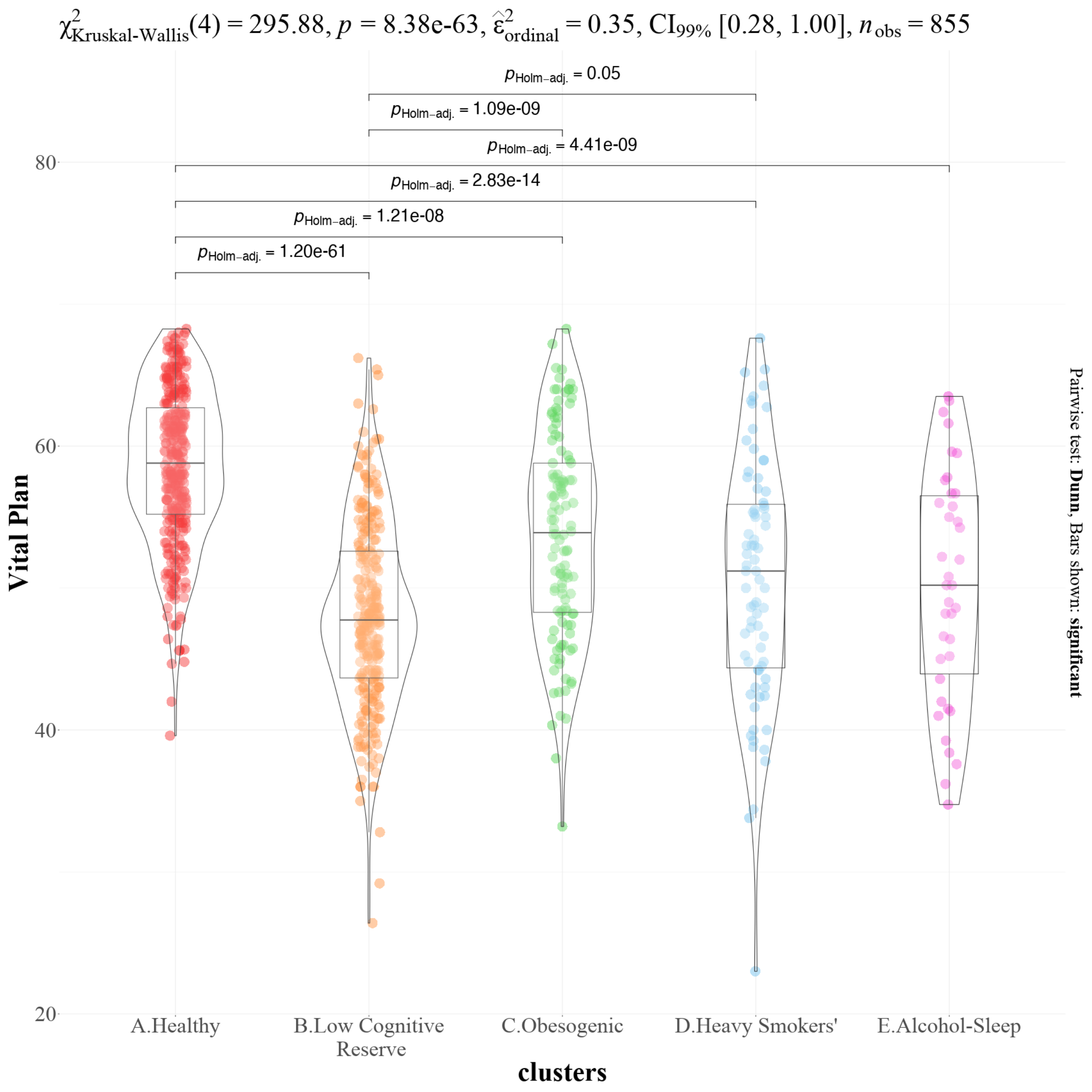

Figure S6. Comparison of Vital Plan mean score across clusters.

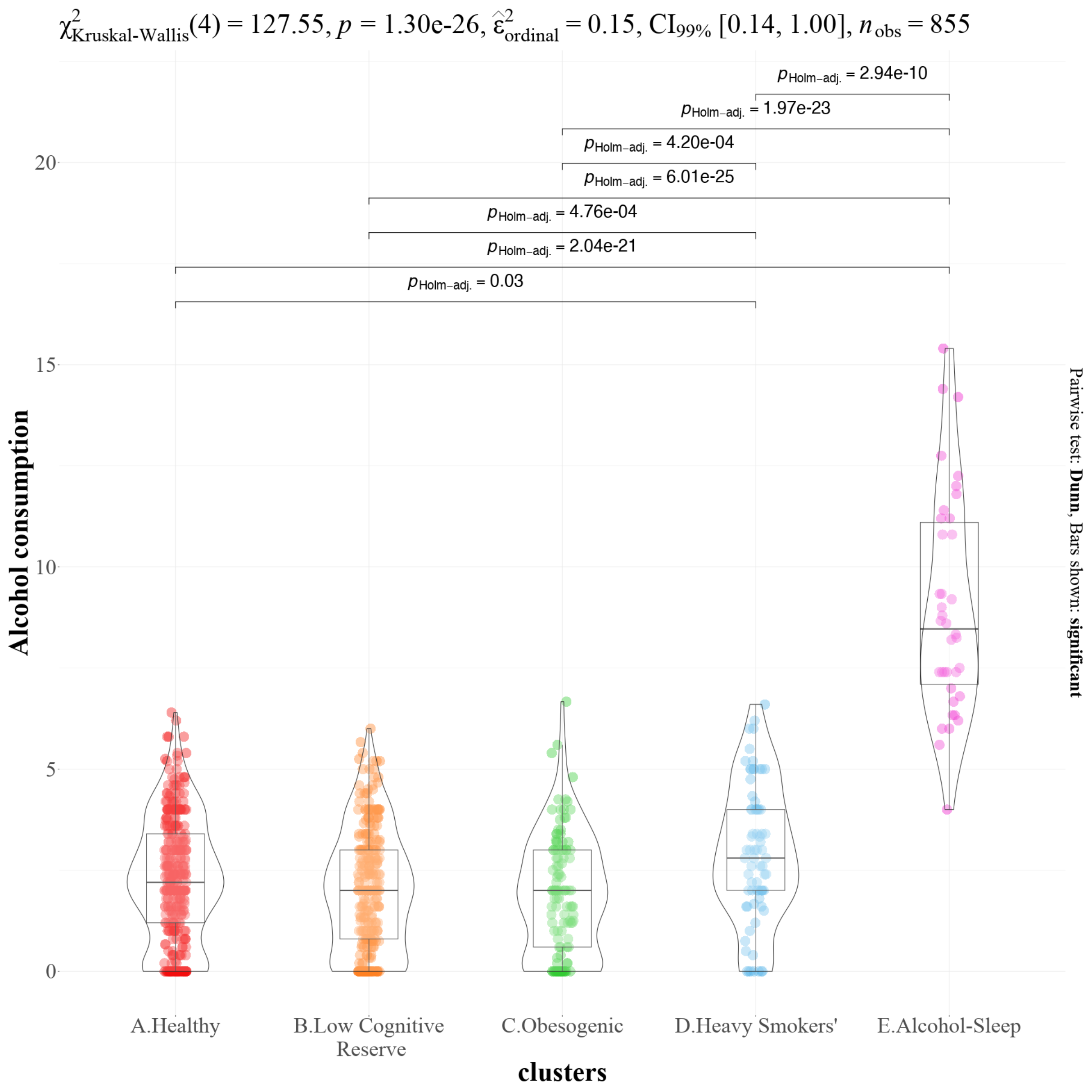

Figure S7. Comparison of Alcohol consumption mean score across clusters.

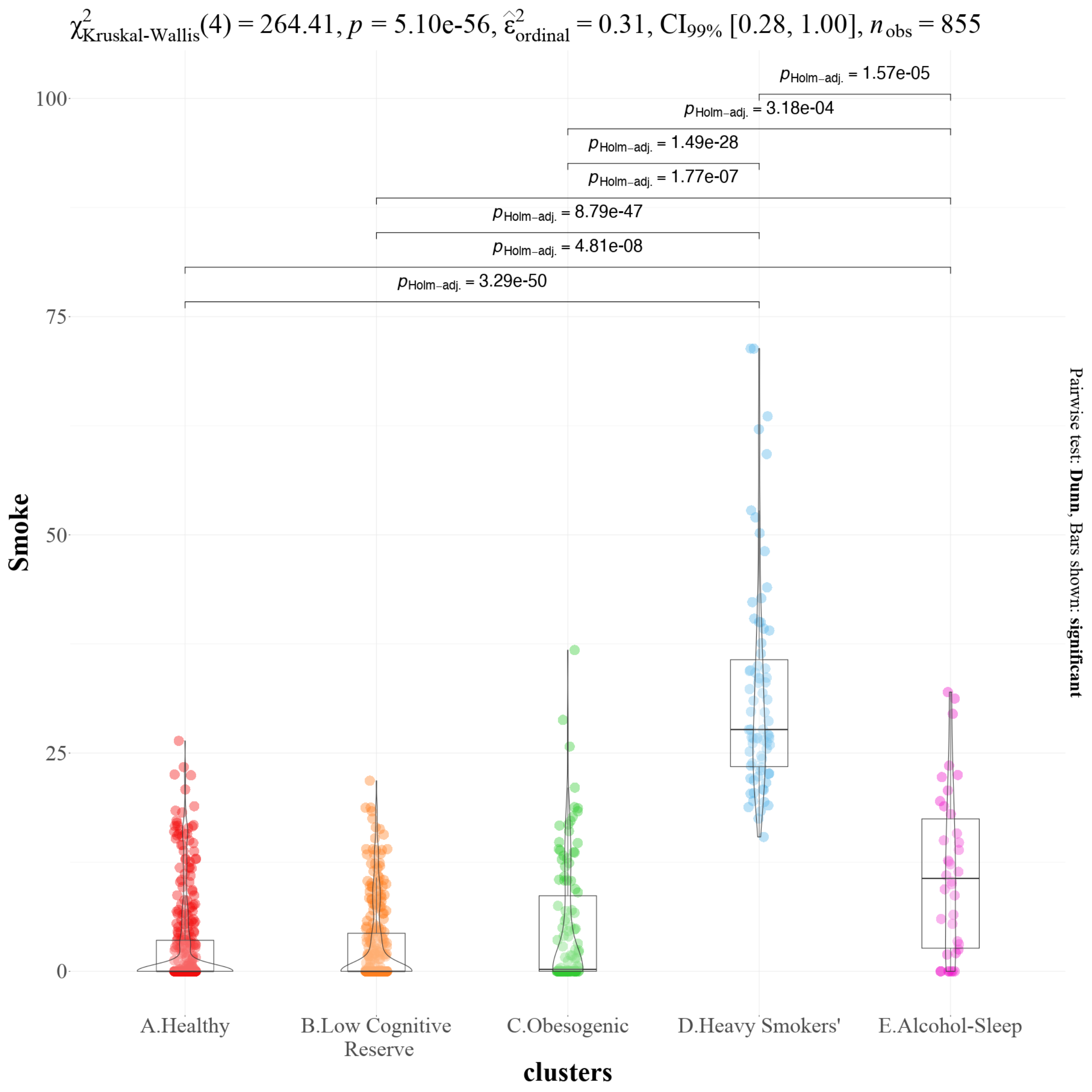

Figure S8. Comparison of tobacco use mean score across clusters.

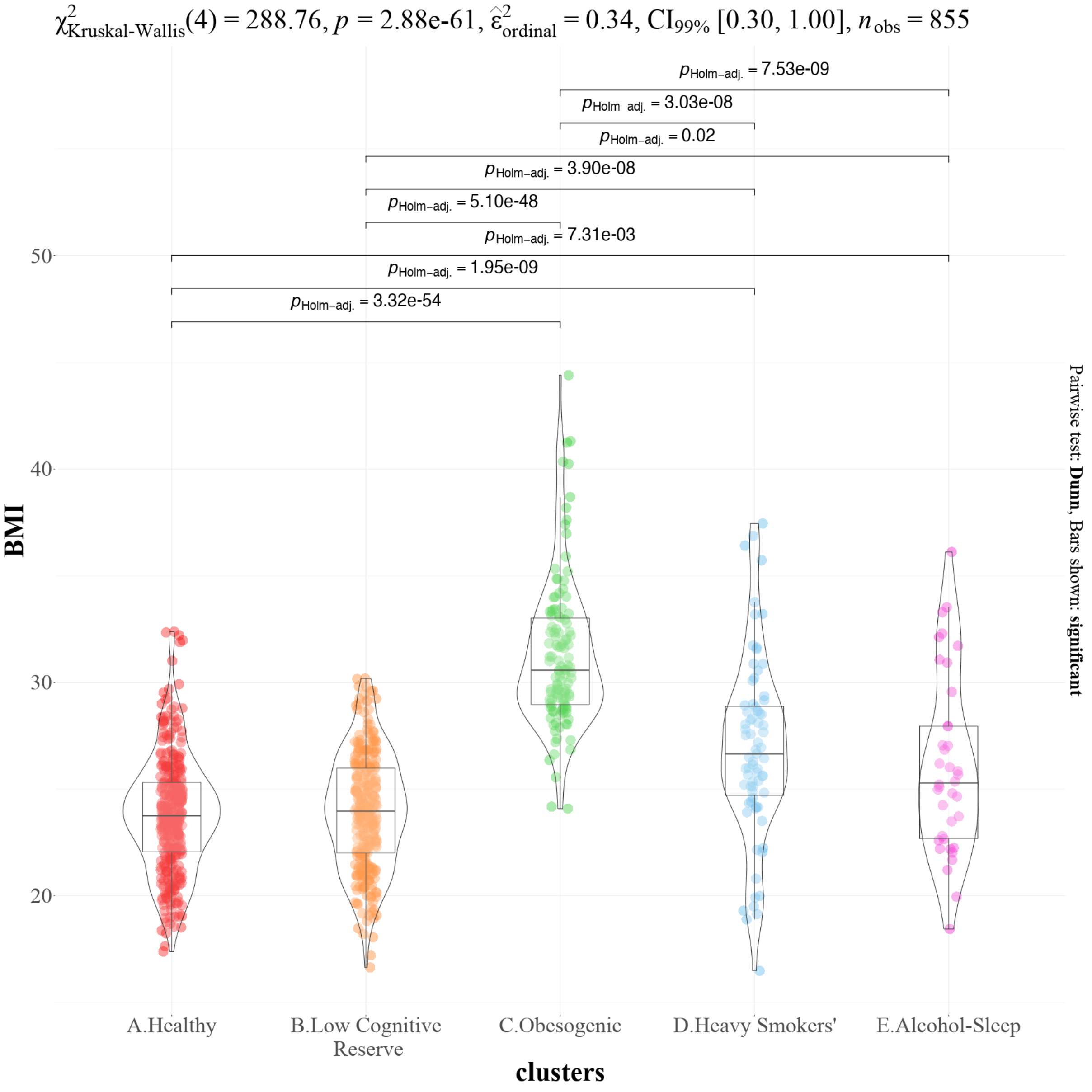

Figure S9. Comparison of BMI mean score across clusters.

Table S4.

Effect sizes and statistical power for the main effects and interactions in ANOVA and linear mixed-effects models.

		**Anova**	
		**Effect size**	**Power**
Main effects		0.0423	0.999
Post Hoc	A-B	0.441	0.999
	A-C	0.281	0.856
	A-D	0.334	0.85
	A-E	0.238	0.246
	B-C	0.16	0.392

	B-D	0.104	0.108
	B-E	0.196	0.464
	C-D	0.056	0.128
	C-E	0.037	0.1
	D-E	0.09	0.212
**Mixed Linear Model**			
Clusters		0.03	0.999
Time		0.007	0.999
Clusters:time Interaction		0.0006	0.36

Note: Power analyses were conducted using the pwr package in R, with effect sizes based on Cohen's d. A= Healthy; B = Low Cognitive Reserve; C = Obesogenic; D = Heavy Smokers; E = Alcohol-Sleep

Table S5.

Variance in education, occupation, and sex explained by CRQ and clusters

Outcome	Model	Predictors	Residual DF	Residual SS	ΔDF	ΔSS	F	p-value
**Education**	**Model 1**	**education ~ CRQ**	3011	752.92				
	**Model 2**	**education ~ CRQ + clusters**	3007	744.37	4	8.55	8.64	<0.001
**Occupation**	**Model 1**	**occupation ~ CRQ**	2973	3246.8				
	**Model 2**	**occupation ~ CRQ + clusters**	2969	3222.7	4	24.1	5.55	<0.001
**Sex**	**Model 1**	**sex ~ CRQ**	3011	660.07				
	**Model 2**	**sex ~ CRQ + clusters**	3007	650.29	4	9.78	11.3	<0.001

Note: CRQ significantly predicts education and occupational level. Clusters add only minor explanatory value beyond CRQ (ΔR^2 ~1.1% for education, ~0.7% for occupation). For sex, which is not included in CRQ, clusters explain additional variance. These results support adjusting for sex, but not for education or occupation, to avoid statistical overadjustment.

Table S6.

Linear regression model predicting ranked outcome (w1_ranked) based on cluster membership, education, sex, and occupation. Cluster A (Healthy) is the reference category. Education is entered as a continuous variable. No interaction terms included.

	**Model 1**				
	**Coefficients**	**Std. Error**	**T Stat**	**P-Value**	
**(Intercept)**	471.99	16.31	28.948	< 2e-16	***
**clustersB**	-98.33	19.67	-5	6.98E-07	***

**clustersC**	-68.19	26	-2.623	0.00887	**
**clustersD**	-89.47	31.15	-2.873	0.00417	**
**clustersE**	-40.77	42.06	-0.969	0.33272	
**sex**	14.81	16.88	0.877	0.38075	

	**Model 2**				
	**Coefficients**	**Std. Error**	**T Stat**	**P-Value**	
**(Intercept)**	-7.687	81.836	-0.094	0.925	
**clustersB**	-75.049	19.774	-3.795	0.00016	***
**clustersC**	-50.474	25.671	-1.966	0.050	*
**clustersD**	-55.846	31.492	-1.773	0.077	.
**clustersE**	-24.457	41.727	-0.586	0.558	
**sex**	14.519	16.625	0.873	0.383	
**education**	95.876	19.119	5.015	0.000	***
**occupation**	5.144	9.076	0.567	0.571	

Note: Linear regression model with 846 observations (9 excluded due to missing data). Residual SE = 237.7; Adjusted R^2 = .072; $F(7, 838) = 10.32$, $p < .001$. A=Healthy; B = Low Cognitive Reserve; C = Obesogenic; D = Heavy Smokers; E = Alcohol-Selex.

Table S7.

Linear mixed-effects model predicting standardized composite scores between Wave 1 and Wave 2. The model includes fixed effects for time, cluster membership, education, occupation and their interactions, with a random intercept for subject (id_user). Education is centered; cluster A (Healthy) is the reference group.

			**Model 1**			
**Fixed effects:**	**Estimate**	**Std. Error**	**df**	**t value**	**Pr(>|t|)**	
**(Intercept)**	1.18E-01	3.33E-02	9.25E+02	3.537	0.000425	***
**Time (Wave2)**	8.33E-02	1.74E-02	6.83E+02	4.793	2.02E-06	***
**Cluster B**	-2.22E-01	4.06E-02	9.66E+02	-5.457	6.16E-08	***
**Cluster C**	-1.57E-01	5.37E-02	9.67E+02	-2.928	0.003495	**
**Cluster D**	-1.62E-01	6.43E-02	9.66E+02	-2.524	0.01175	*
**Cluster E**	-1.14E-01	8.68E-02	9.64E+02	-1.309	0.190981	

**sex**	3.68E-03	3.37E-02	8.47E+02	0.109	0.913055	
**Time × Cluster B**	3.34E-02	2.64E-02	6.86E+02	1.269	0.204832	
**Time × Cluster C**	6.66E-02	3.42E-02	6.82E+02	1.947	0.051889	.
**Time × Cluster D**	-3.40E-04	4.15E-02	6.85E+02	-0.008	0.993463	
**Time × Cluster E**	3.92E-02	5.44E-02	6.81E+02	0.719	0.472249	

		**Model 2**				
**Fixed effects:**	**Estimate**	**Std. Error**	**df**	**t value**	**Pr(>|t|)**	
**(Intercept)**	-8.58E-01	1.64E-01	8.49E+02	-5.25	1.93E-07	***
**Time (Wave2)**	8.39E-02	1.74E-02	6.79E+02	4.824	1.74E-06	***
**Cluster B**	-1.75E-01	4.07E-02	9.54E+02	-4.307	1.83E-05	***
**Cluster C**	-1.23E-01	5.29E-02	9.60E+02	-2.313	0.0209	*
**Cluster D**	-9.47E-02	6.49E-02	9.55E+02	-1.46	0.1447	
**Cluster E**	-8.37E-02	8.60E-02	9.58E+02	-0.973	0.3307	
**Education (years)**	1.96E-01	3.81E-02	8.44E+02	5.144	3.35E-07	***
**sex**	1.84E-03	3.31E-02	8.37E+02	0.055	0.9558	
**occupation**	9.23E-03	1.81E-02	8.36E+02	0.511	0.6097	
**Time × Cluster B**	3.38E-02	2.64E-02	6.82E+02	1.283	0.2	
**Time × Cluster C**	6.30E-02	3.43E-02	6.78E+02	1.838	0.0665	.
**Time × Cluster D**	8.15E-04	4.17E-02	6.80E+02	0.02	0.9844	
**Time × Cluster E**	2.26E-02	5.51E-02	6.77E+02	0.409	0.6825	

*Note: Model fitted using REML. Random intercept for subject (id_user). Number of observations: 1,521; subjects: 855. Significance codes: *** p < .001, ** p < .01, * p < .05, . p < .10. A=Healthy; B = Low Cognitive Reserve; C = Obesogenic; D = Heavy Smokers; E = Alcohol-Sleep.*

Table S8.

Linear mixed-effects model predicting standardized memory scores. The model includes fixed effects for time, cluster membership, education, sex, occupation and their interactions with time. Cluster A (Healthy) is the reference group. A random intercept was included for participant ID.

			**Model 1**			
**Fixed effects:**	**Estimate**	**Std. Error**	**df**	**t value**	**Pr(>|t|)**	
**(Intercept)**	0.01114	0.05143	998.49201	0.217	0.82853	
**Time (Wave2)**	0.22709	0.0377	691.0055	6.024	2.77E-09	***
**Cluster B**	-0.27388	0.06347	1081.7537	-4.315	1.74E-05	***
**Cluster C**	-0.22131	0.08394	1083.38142	-2.637	0.00849	**
**Cluster D**	-0.2614	0.10052	1081.94615	-2.6	0.00944	**
**Cluster E**	-0.16906	0.13564	1078.72661	-1.246	0.21291	
**sex**	0.25992	0.05083	830.29327	5.114	3.92E-07	***
**Time × Cluster B**	0.09568	0.05711	697.32085	1.675	0.0943	.
**Time × Cluster C**	0.11784	0.07424	689.54693	1.587	0.11291	

**Time × Cluster D**	0.13636	0.08992	694.93625	1.517	0.12984	
**Time × Cluster E**	0.12659	0.11823	687.21433	1.071	0.28468	

			**Model 2**			
**Fixed effects:**	**Estimate**	**Std. Error**	**df**	**t value**	**Pr(>|t|)**	
**(Intercept)**	-1.44196	0.24914	843.24647	-5.788	1.01E-08	***
**Time (Wave2)**	0.2265	0.03788	687.22571	5.98	3.58E-09	***
**Cluster B**	-0.22191	0.06406	1067.70267	-3.464	0.000553	***
**Cluster C**	-0.18487	0.08341	1078.15828	-2.216	0.026871	*
**Cluster D**	-0.16259	0.10208	1069.6602	-1.593	0.111509	
**Cluster E**	-0.14994	0.13545	1074.87161	-1.107	0.268561	
**Education (years)**	0.31984	0.05803	833.1227	5.512	4.74E-08	***
**sex**	0.26064	0.05024	820.0577	5.188	2.69E-07	***
**occupation**	-0.02881	0.02741	818.19603	-1.051	0.293533	
**Time × Cluster B**	0.09508	0.05732	694.10306	1.659	0.097624	.
**Time × Cluster C**	0.1154	0.07465	686.60123	1.546	0.122607	
**Time × Cluster D**	0.13727	0.09074	690.49111	1.513	0.130785	
**Time × Cluster E**	0.11801	0.12008	684.92386	0.983	0.326097	

*Note: Linear mixed-effects model with random intercept for subject (id_user). Model fit using REML on 1,521 observations from 855 participants. Significance codes: *** p < .001, ** p < .01, * p < .05, . p < .10. A=Healthy; B = Low Cognitive Reserve; C = Obesogenic; D = Heavy Smokers; E = Alcohol-Sleep.*

Table S9.

Linear mixed-effects model predicting standardized executive function scores. The model includes fixed effects for time, cluster membership, education, sex, occupation and time × cluster interactions. Cluster A (Healthy) is the reference category. A random intercept was included for each participant.

			**Model 1**			
**Fixed effects:**	**Estimate**	**Std. Error**	**df**	**t value**	**Pr(>|t|)**	
**(Intercept)**	0.16353	0.03894	1001.08002	4.199	2.92E-05	***
**Time (Wave2)**	0.04033	0.02714	709.07432	1.486	0.1377	
**Cluster B**	-0.23273	0.04795	1074.46726	-4.853	1.39E-06	***
**Cluster C**	-0.12701	0.06341	1075.90715	-2.003	0.0454	*
**Cluster D**	-0.09697	0.07594	1074.63749	-1.277	0.2019	
**Cluster E**	-0.04061	0.10248	1071.79016	-0.396	0.692	
**sex**	-0.09396	0.03868	853.24855	-2.429	0.0153	*
**Time × Cluster B**	0.0262	0.04112	714.82404	0.637	0.5243	
**Time × Cluster C**	0.07148	0.05344	707.74385	1.338	0.1815	
**Time × Cluster D**	-0.01063	0.06474	712.65332	-0.164	0.8696	

**Time × Cluster E**	-0.02197	0.0851	705.61538	-0.258	0.7964	

			Model 2			
**Fixed effects:**	**Estimate**	**Std. Error**	**df**	**t value**	**Pr(>|t|)**	
**(Intercept)**	-0.62057	0.19061	862.81914	-3.256	0.001175	**
**Time (Wave2)**	0.0417	0.02723	703.73279	1.532	0.126095	
**Cluster B**	-0.19279	0.04865	1056.88766	-3.963	7.91E-05	***
**Cluster C**	-0.09397	0.06332	1066.02042	-1.484	0.138119	
**Cluster D**	-0.04397	0.07752	1058.6125	-0.567	0.570685	
**Cluster E**	-0.01314	0.10284	1063.14209	-0.128	0.898324	
**Education (years)**	0.15366	0.04441	853.9482	3.46	0.000567	***
**sex**	-0.09697	0.03847	842.24883	-2.521	0.011897	*
**occupation**	0.01396	0.02099	840.57817	0.665	0.506093	
**Time × Cluster B**	0.02281	0.04122	709.90914	0.553	0.580187	
**Time × Cluster C**	0.06075	0.05366	703.17154	1.132	0.257944	
**Time × Cluster D**	-0.01116	0.06524	706.66645	-0.171	0.864251	
**Time × Cluster E**	-0.03859	0.08631	701.65754	-0.447	0.654921	

*Note: Linear mixed-effects model with a random intercept for participant (id_user). Model fit using REML. Observations: 1,521 from 855 participants. Significance codes: *** p < .001, ** p < .01, * p < .05. A=Healthy; B = Low Cognitive Reserve; C = Obesogenic; D = Heavy Smokers; E = Alcohol-Sleep.*

Table S10.

Linear mixed-effects model predicting standardized processing speed. The model includes fixed effects for time, cluster membership, education, occupation and their interactions, with a random intercept for subject (id_user). Education is centered; cluster A (Healthy) is the reference group.

			Model 1			
**Fixed effects:**	**Estimate**	**Std. Error**	**df**	**t value**	**Pr(>|t|)**	
**(Intercept)**	4.51E-02	5.43E-02	9.72E+02	0.831	0.40608	
**Time (Wave2)**	-5.37E-02	3.53E-02	6.95E+02	-1.524	0.12807	
**Cluster B**	-1.72E-01	6.66E-02	1.04E+03	-2.574	0.0102	*
**Cluster C**	-2.12E-01	8.81E-02	1.04E+03	-2.403	0.01644	*
**Cluster D**	-2.49E-01	1.06E-01	1.04E+03	-2.364	0.01828	*
**Cluster E**	-2.06E-01	1.42E-01	1.03E+03	-1.448	0.14798	
**sex**	1.64E-01	5.42E-02	8.46E+02	3.023	0.00258	**
**Time × Cluster B**	-5.43E-03	5.34E-02	7.00E+02	-0.102	0.91915	
**Time × Cluster C**	6.22E-02	6.94E-02	6.94E+02	0.896	0.37049	
**Time × Cluster D**	-9.72E-02	8.41E-02	6.98E+02	-1.156	0.24818	
**Time × Cluster E**	8.34E-02	1.11E-01	6.92E+02	0.754	0.45085	

		Model 2				
**Fixed effects:**	**Estimate**	**Std. Error**	**df**	**t value**	**Pr(>|t|)**	
**(Intercept)**	1.39E-01	4.47E-02	1.04E+03	3.102	0.00197	**
**Time (Wave2)**	-5.34E-02	3.53E-02	6.94E+02	-1.515	0.13025	
**Cluster B**	-1.89E-01	6.67E-02	1.04E+03	-2.831	0.00473	**
**Cluster C**	-2.21E-01	8.84E-02	1.04E+03	-2.495	0.01273	*
**Cluster D**	-2.75E-01	1.06E-01	1.04E+03	-2.607	0.00926	**
**Cluster E**	-2.65E-01	1.42E-01	1.04E+03	-1.873	0.05131	*
**Education (years)**	0.23588	0.06217	845.04151	3.794	0.000159	***
**sex**	0.16851	0.05388	834.66533	3.128	0.001824	**
**occupation**	0.0245	0.0294	833.18179	0.833	0.404843	
**Time × Cluster B**	-6.20E-03	5.35E-02	6.99E+02	-0.116	0.90773	
**Time × Cluster C**	6.32E-02	6.95E-02	6.93E+02	0.91	0.36291	
**Time × Cluster D**	-9.90E-02	8.42E-02	6.97E+02	-1.176	0.23989	
**Time × Cluster E**	8.51E-02	1.11E-01	6.91E+02	0.77	0.44178	

*Note: Model fitted using REML. Random intercept for subject (id_user). Number of observations: 1,521; subjects: 855. Significance codes: *** p < .001, ** p < .01, * p < .05, . p < .10. A=Healthy; B = Low Cognitive Reserve; C = Obesogenic; D = Heavy Smokers; E = Alcohol-Sleep.*

References

Bittner, N., Jockwitz, C., Franke, K., Gaser, C., Moebus, S., Bayen, U. J., et al. (2021). When your brain looks older than expected: combined lifestyle risk and BrainAGE. *Brain Struct. Funct.* 226, 621–645. doi: 10.1007/s00429-020-02184-6

Conigrave, K. M., Saunders, J. B., and Reznik, R. B. (1995). Predictive capacity of the AUDIT questionnaire for alcohol-related harm. *Addiction* 90, 1479–1485. doi: 10.1111/j.1360-0443.1995.tb02810.x

Craig, C. L., Marshall, A. L., Sjöström, M., Bauman, A. E., Booth, M. L., Ainsworth, B. E., et al. (2003). International Physical Activity Questionnaire: 12-Country Reliability and Validity. *Med. Sci. Sports Exerc.* 35, 1381–1395. doi: 10.1249/01.MSS.0000078924.61453.FB

Duriez, Q., Crivello, F., and Mazoyer, B. (2014). Sex-related and tissue-specific effects of tobacco smoking on brain atrophy: assessment in a large longitudinal cohort of healthy elderly. *Front. Aging Neurosci.* 6. doi: 10.3389/fnagi.2014.00299

Franklin, T. R., Wetherill, R. R., Jagannathan, K., Johnson, B., Mumma, J., Hager, N., et al. (2014). The Effects of Chronic Cigarette Smoking on Gray Matter Volume: Influence of Sex. *PLoS ONE* 9, e104102. doi: 10.1371/journal.pone.0104102

Jenkins, C. D., Stanton, B. A., Niemcryk, S. J., and Rose, R. M. (1988). A scale for the estimation of sleep problems in clinical research. *J. Clin. Epidemiol.* 41, 313–321. doi: 10.1016/0895-4356(88)90138-2

Karama, S., Ducharme, S., Corley, J., Chouinard-Decorte, F., Starr, J. M., Wardlaw, J. M., et al. (2015). Cigarette smoking and thinning of the brain's cortex. *Mol. Psychiatry* 20, 778–785. doi: 10.1038/mp.2014.187

Lubben, J. E. (1988). Assessing social networks among elderly populations. *Fam. Community Health J. Health Promot. Maint.* 11, 42–52. doi: 10.1097/00003727-198811000-00008

Rami, L., Valls-Pedret, C., Bartrés-Faz, D., Caprile, C., Solé-Padullés, C., Castellvi, M., et al. (2011). Cognitive reserve questionnaire. Scores obtained in a healthy elderly population and in one with Alzheimer's disease. *Rev. Neurol.* 52, 195–201.

Ryff, C. D. (1995). Psychological Well-Being in Adult Life. *Curr. Dir. Psychol. Sci.* 4, 99–104. doi: 10.1111/1467-8721.ep10772395

Schröder, H., Fitó, M., Estruch, R., Martínez-González, M. A., Corella, D., Salas-Salvadó, J., et al. (2011). A Short Screener Is Valid for Assessing Mediterranean Diet Adherence among Older Spanish Men and Women. *J. Nutr.* 141, 1140–1145. doi: 10.3945/jn.110.135566
